# ﻿An annotated and illustrated identification guide to common mesophotic reef sponges (Porifera, Demospongiae, Hexactinellida, and Homoscleromorpha) inhabiting Flower Garden Banks National Marine Sanctuary and vicinities

**DOI:** 10.3897/zookeys.1161.93754

**Published:** 2023-05-11

**Authors:** Maria Cristina Díaz, Marissa Nuttall, Shirley A. Pomponi, Klaus Rützler, Sarah Klontz, Christi Adams, Emma L. Hickerson, G. P. Schmahl

**Affiliations:** 1 Harbor Branch Oceanographic Institute, Florida Atlantic University, Fort Pierce, FL, USA Florida Atlantic University Fort Pierce United States of America; 2 Flower Garden Banks National Marine Sanctuary, Galveston, TX, USA Flower Garden Banks National Marine Sanctuary Galveston United States of America; 3 CPC Inc, Galveston, TX, USA CPC Inc Galveston United States of America; 4 National Museum of Natural History, Smithsonian Institution, Washington D.C., USA National Museum of Natural History, Smithsonian Institution Waschngton DC United States of America; 5 Genetic Disease Research Branch, NHGRI, NIH, Bethesda, MD, USA Genetic Disease Research Branch, NHGRI, NIH Bethesda United States of America

**Keywords:** Algal reefs, biodiversity, Gulf of Mexico, mesophotic reefs, Porifera, sponges

## Abstract

Sponges are recognized as a diverse and abundant component of mesophotic and deep-sea ecosystems worldwide. In Flower Garden Banks National Marine Sanctuary region within the northwestern Gulf of Mexico, sponges thrive among diverse biological and geological habitats between 16–200+ m deep (i.e., coral reefs and communities, algal nodules, and coralline algae reefs, mesophotic reefs, patch reefs, scarps, ridges, soft substrate, and rocky outcrops). A synoptic guide is presented, developed by studying common sponge species in the region, through direct sampling and *in-situ* photographic records. A total of 64 species is included: 60 are Demospongiae (14 orders), two are Hexactinellida (one order), and two are Homoscleromorpha (one order). Thirty-four taxa are identified to species and 13 were identified to have affinity with, but were not identical to, a known species. Fifteen taxa could only be identified to genus level, and the species remain as uncertain (incerta sedis), with the potential to represent new species or variants of known species. One specimen received only a family assignation. This study extends geographic or mesophotic occurrence data for eleven known species and includes several potentially new species. This work improves our knowledge of Gulf of Mexico sponge biodiversity and highlights the importance of the region for scientists and resource managers.

## ﻿Introduction

**Figure 1.**
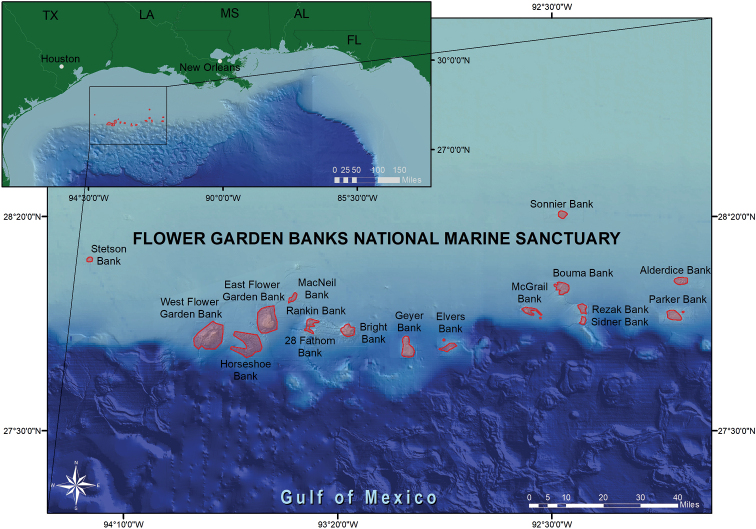
Map of Flower Garden Banks National Marine Sanctuary.

Flower Garden Banks National Marine Sanctuary consists of portions of 17 topographic features in the northwestern Gulf of Mexico. The reefs and banks occur along the continental shelf, from 70–120 miles off the coast of Texas and Louisiana (Fig. 1), range in depth from 16–220 m, and harbor coral reefs, coral communities, coralline algae reefs, rhodolith beds, and deep mesophotic communities. Extensive remotely operated vehicle explorations within the region have been conducted during the past 30 years by National Oceanic and Atmospheric Administration’s (NOAA’s) Flower Garden Banks National Marine Sanctuary (FGBNMS) and partners, including NOAA’s Deep-Sea Coral Research and Technology Program (ONMS 2016). More than 50,000 geo-referenced images, 900 hours of video, and 38 annotation logs have been collected during those expeditions, and multiple databases have been produced. This prior work discovered that the region consists of a series of unique and interconnected habitats of banks, coral reefs, patch reefs, scarps, and ridges, featuring algal dominated areas, soft substrate features, mesophotic and deep coral communities and rocky outcrops (Schmahl et al. 2008). A comprehensive review of the biology and ecology of coral reefs, coral communities, and mesophotic habitats in this region, including the area within the sanctuary boundaries, have documented four major reef-related habitats: i) a “coral reef zone” from approximately 0–70 m that includes the actively accreting hermatypic coral assemblages and a shallow mesophotic coral community, ii) a “coral community zone” that occurs primarily in depths less than 50 m where hermatypic coral species are present at low densities but are not dominant, iii) a “coralline algae” or mid-mesophotic zone occurring in depths 60–120 m and characterized by rocky outcrops with a predominance of crustose coralline algal nodules, and iv) a lower mesophotic reef occurring between 90–200 m, characterized by antipatharian and alcyonarian corals, crinoids, bryozoans, sponges, azooxanthellate branching corals, and small, solitary hard corals (Schmahl et al. 2008; Semmler et al. 2016; Nuttall et al. 2022). These complex underwater features provide feeding areas, spawning sites and habitat for critical life history stages for a variety of reef organisms (Schmahl et al. 2008).

Sponges are recognized as a diverse and abundant component of mesophotic and deep-sea ecosystems (Pomponi et al. 2019, Slattery et al. 2017; Schmahl et al. 2012), and in 2017 the development of field guides was identified as a priority in NOAA’s Science Plan for the Southeast Deep Coral Initiative (Wagner et al. 2017). Hickerson and Schmahl (2012) created a quick reference photo guide poster for 37 species studied and identified by KR, SWK, and CA that had been documented during exploratory dives between 50 and 110 m deep within the northwestern Gulf of Mexico. More surveys have been conducted since 2012, resulting in additional sponge specimens and imagery that required further investigation. These expeditions investigated areas that were under consideration for sanctuary expansion and officially became part of the sanctuary in 2021 (15 CFR Part 922 – Subpart L, 2021). Twenty-seven morphospecies of emergent sponges from the Classes Demospongiae and Hexactinellida and an unaccounted number of thin encrusting species were documented during an expedition in 2019. This study expanded the recognized sponge biodiversity of Flower Garden Banks National Marine Sanctuary region by 17 species (https://flowergarden.noaa.gov/about/spongelist.html) and includes at least six species potentially new to science: *Pleraplysilla* sp. 2, *Geodia* sp. 1, *Cinachyrella* sp. 1, *Auletta* sp. 1, *Petrosia* sp. 1, and *Xestospongia* sp. 1.

The major goal of this study was to update current knowledge of Porifera biodiversity from mesophotic depths at the sanctuary region and to promote this knowledge among major stakeholders. We have developed a synoptic identification guide that can be used by a wide range of end-users (i.e., marine scientists and students, conservationists, environmental managers, naturalists, recreational divers, etc.). This identification guide summarizes the current taxonomic status and distinct features for 63 species distinguished of the common sponge species encountered in the region. This work will improve our knowledge of sponge biodiversity in the Gulf of Mexico and enhance studies of sponges from mesophotic and deep-water ecosystems in the region. Furthermore, this first illustrated guide to species at mesophotic depths in the northwestern Gulf of Mexico will facilitate the comparisons with recently studied mesophotic sponge fauna from other deep mesophotic habitats occurring at Pulley Ridge in the southeast Gulf of Mexico (MCD unpublished data), Cuba (Reed et al. 2018; Díaz et al. 2019) and southeast USA Deep Ecosystems in Marine Protected Areas (Díaz et al. 2021; Reed et al. 2021). The potential discovery of new species and its importance will be discussed herein.

## ﻿Materials and methods

### ﻿Area of study

This study focused on topographic features in the northwestern Gulf of Mexico in and around Flower Garden Banks National Marine Sanctuary located on the continental shelf edge in the northwestern Gulf of Mexico in the USA (Fig. 1). Samples presented in this study were collected both within and adjacent to the sanctuary boundaries and occurred within one of the six habitats (coral reefs, coral communities, algal nodules, coralline algae reefs, lower mesophotic reefs, and soft substrates) described by Schmahl et al. (2008) and Semmler et al. (2016). The waters in the region are typically oligotrophic, warm tropical water that is transported from the Caribbean into the eastern Gulf of Mexico via the Loop Current and travels to the western Gulf through the action of spin-off eddies (see Schmahl et al. 2008: fig. 6.6). The offshore location (60–130 miles off the continental coast) of these habitats typically separates them from turbid, brackish, coastal waters and the influence of coastal runoff and nearshore eutrophication. However, sporadic coastal water intrusion events have been documented in the region (Kealoha et al. 2020).

### ﻿Collection methods and data

**Figure 2.**
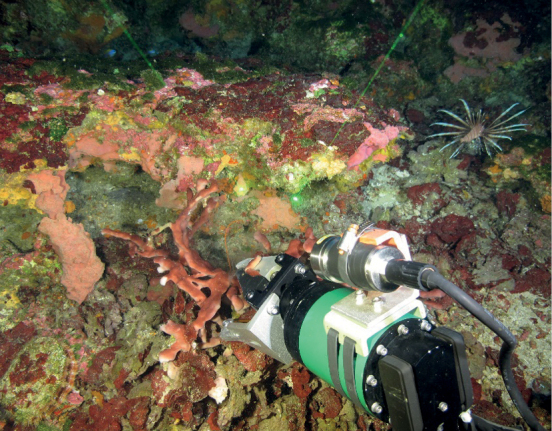
Sample collection DFH33-542A using a manipulator mounted on an ROV. Green scale lasers, 10 cm apart seen in the field of view, were used to estimate the size of the specimen.

Collections were made using one of three remotely operated vehicles (ROV), including Phantom S2, owned and operated by University of North Carolina at Wilmington (UNCW) Undersea Vehicle Program, MOHAWK, owned by the National Marine Sanctuary Foundation and operated by UNCW Undersea Vehicle Program, and YOGI, owned and operated by the Global Foundation for Ocean Exploration. Specimens were photographed in situ using a variety of digital still cameras with scale lasers in the field of view set at 10 cm (Fig. 2). Sponges were collected using a manipulator on the ROV and either brought directly to the surface in the manipulator or placed in a sample box mounted on the ROV. Once on the surface, sponges were photographed in the lab using a digital still camera prior to preservation. Sample metadata, including location (latitude and longitude), depth, and habitat were recorded into a Microsoft Excel database archived at Flower Garden Banks National Marine Sanctuary offices in Galveston, TX. Specimens were either preserved in 95% ethanol, and occasionally in 10% formalin in sea water for histological evaluation when specimens were potential new species. Samples were stored at the Flower Garden Banks National Marine Sanctuary offices in Galveston, TX, except for samples collected in 2019, which are archived at Florida Atlantic University – Harbor Branch Oceanographic Institute, Marine Biotechnology Reference Collection (http://hboi-marine-biomedical-and-biotechnology-reference-collection.fau.edu/app/data-portal). Suppl. material 1 lists all species included in this guide, location of observations, and their abundance at each site.

### ﻿Species data and morphological characterization

Each species within this guide is represented by an *in-situ* image, the lowest available scientific name, species author/date, higher taxonomy, depth, and sample number (indicated in the figure legend). The species data are divided in six sections: “Diagnostic features” describes distinctive morphologic features; Similar species with which it might be confused; “Distribution and abundance” refers to overall regional distribution from the World Porifera Database and other recent references (Pomponi et al. 2019) indicating countries and/or regions where the species occur as well as the number of sites within the sanctuary (i.e., East Flower Garden Bank, Geyer Bank, etc.) in which the species was observed and a qualitative range of abundances (Suppl. material 1); “Ecology” mentions the habitat(s) where each species occurs; “ID” indicates the individual(s) who identified the sample by their initials, and “References” provides literature where the reader can get a more detailed description including other characters such as spicules, skeletal architecture, or genetic information.

Fifty-two of the 64 species included on this study were identified by the analysis of one or more samples. Therefore, the majority (~ 84%) of the identifications were confirmed by evaluation of skeletal morphology (skeleton type, size, and architecture) as well as features of the external morphology. Skeleton analysis was carried out using methods described in Díaz and Pomponi (2018) but using a rapid tissue digestion in bleach instead of nitric acid. The taxonomic assignation for each morphotype reflects the most current classification of the World Porifera Database (de Voogd et al. 2023). The occurrence and qualitative estimate of abundance was made within an approximate area of 259 sq. miles (the core biological zone that FGB uses to bound explorations; Office of National Marine Sanctuaries 2020). The occurrence at each site is characterized according to the approximate number of specimens observed as: Single (**S**) if only one specimen was observed, Few (**F**) 2–10 specimens, Many (**M**) 10–100 specimens, and Abundant (**A**) more than 100 specimens.

We use the same criteria to describe the external morphology as in the recent guide to sponges from deep marine protected areas from the southeastern USA (Díaz et al. 2021). Each morphospecies is characterized by its external appearance (shape, surface features, color patterns, oscula). Sponge shapes are described according to their 3-dimensional growth as encrusting (thin or thick but following the contour of the substrate) or massive (the sponge develops away from the substrate). The shape may represent a particular geometry (tubular, cylindrical, globular or sub-globular, cup, or fan) or a particular pattern (bushy, arborescent, amorphous). “Surface” refers to details of the outer appearance; it may be smooth, convoluted, rugose, velvet, porous, or have projections that might be cone-shaped (conulose), hairy (hispid), or with digitated hollow blind projections (fistulose). The smaller, incurrent water apertures (ostia) may be aggregated in papillae, clumps, or porocalices. The larger excurrent water apertures usually represent oscula or pseudo-oscula and are described by morphology (shape, presence of a membrane or collar, etc.), abundance (sparse, common, or abundant), location (apical, regularly distributed, in clumps, on a sieve plate), size (diameter, measured when they are visible), and the presence and nature of a membrane (flush, elevated, collar, transparent, colored). The sponge consistency, ranging from soft, compressible, cartilaginous, crumbly, or hard, is also a useful feature to characterize sponge species. These are useful details to characterize and distinguish the majority of sponge species. Definition of these descriptive terms for sponge external morphology can be found in the Sponge Thesaurus (Boury-Esnault and Rützler 1997).

### ﻿Species checklist terms and abbreviations

**aff.** affinis; the species might appear similar but is not that species. Implies a higher degree of uncertainty compared to cf. (Sigovini et al. 2016);

**cf.** confer; to be compared with. Indicates that most of the diagnostic characters correspond to a given species, but some characters are unclear. The identification is provisional but is likely to be definitive after comparing with reference material or consulting a specialist of the taxon (Sigovini et al. 2016);

**FGBNMS** Flower Garden Banks National Marine Sanctuary;

**GOM** Gulf of Mexico;

**sp. nov.** new species to science. Specimen has unique characters that can support our interpretation about its distinct and unique identity;

**spp.** species in plural. Refers to multiple species from a particular genus.

## ﻿Results

### ﻿Taxonomic scope

**Figure 3.**
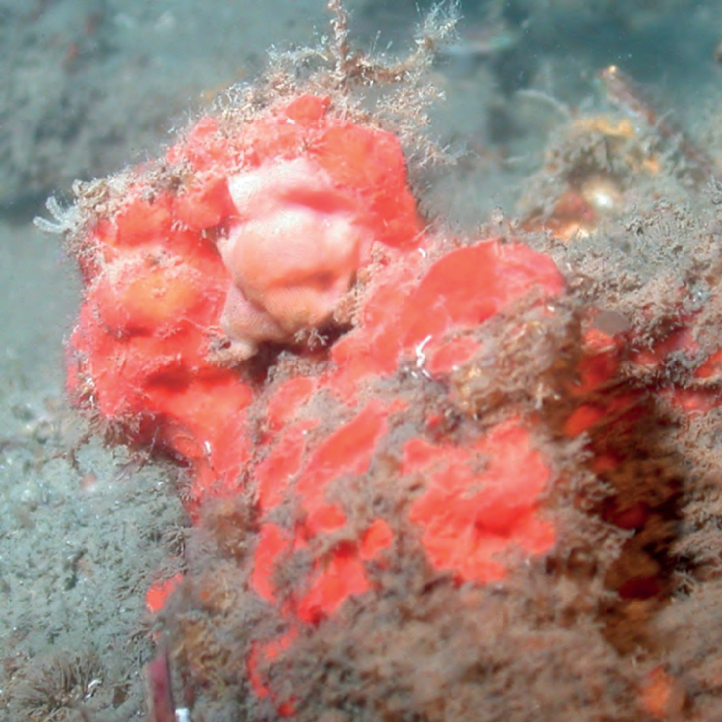
Agelascf.citrina, 50 m deep. Sample DFH9-7E.

**Figure 4.**
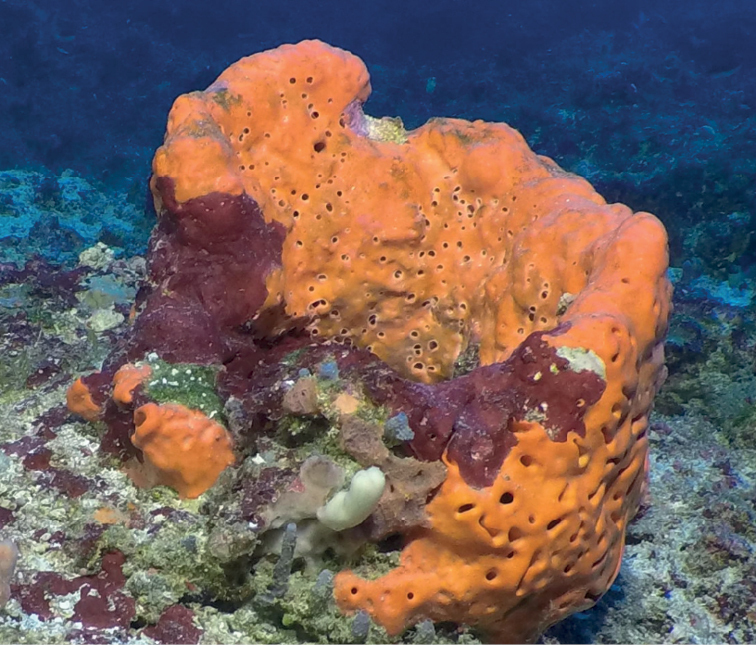
*Agelasclathrodes*, 61 m deep. Photo code YG1901L3_IMG_20190831T212309Z.

**Figure 5.**
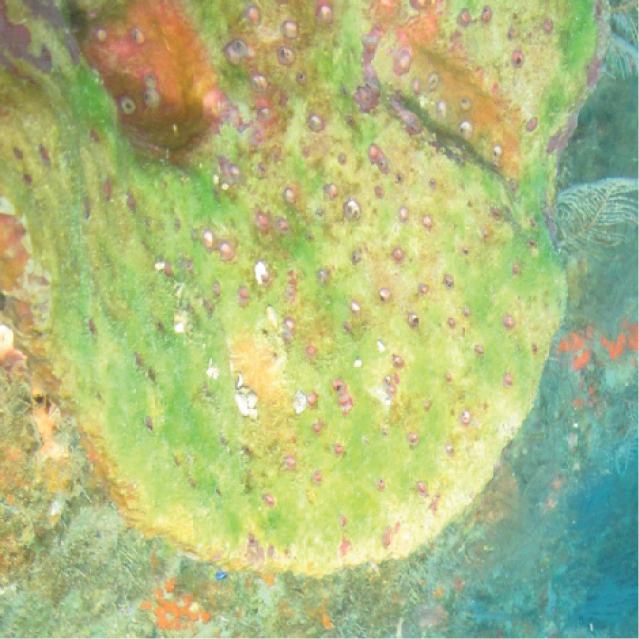
*Agelasdilatata*, 46 m deep. Photo code SP-49.

**Figure 6.**
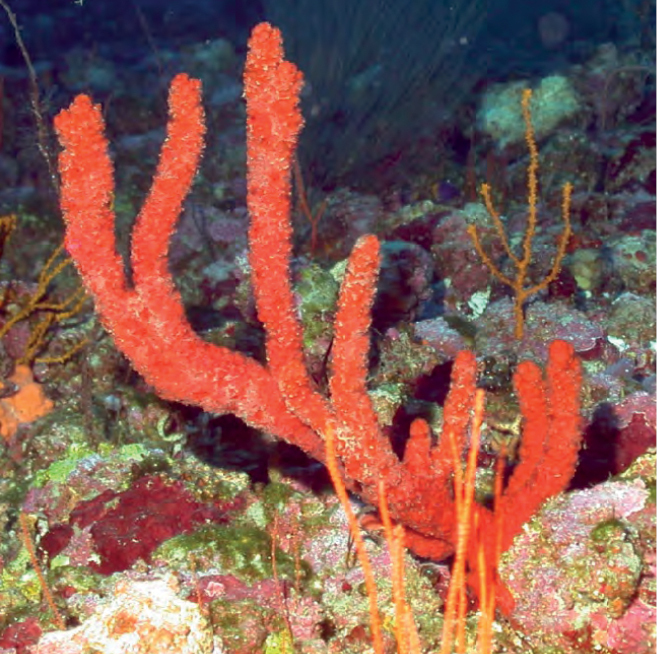
Ptilocauliscf.walpersii, 60 m deep, Photo code SP-01.

**Figure 7.**
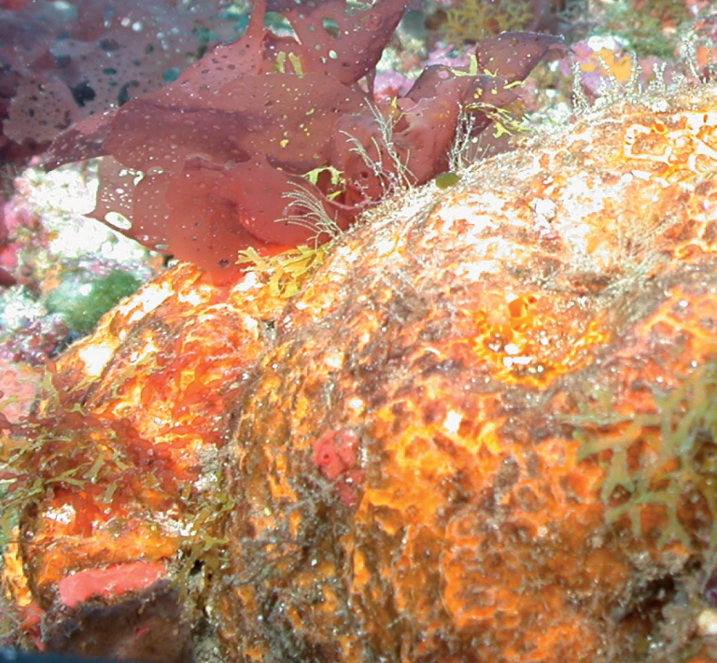
*Myrmekiodermagyroderma*. 60–72 m deep. Samples DFH9-11A, DFH9-5B.

**Figure 8.**
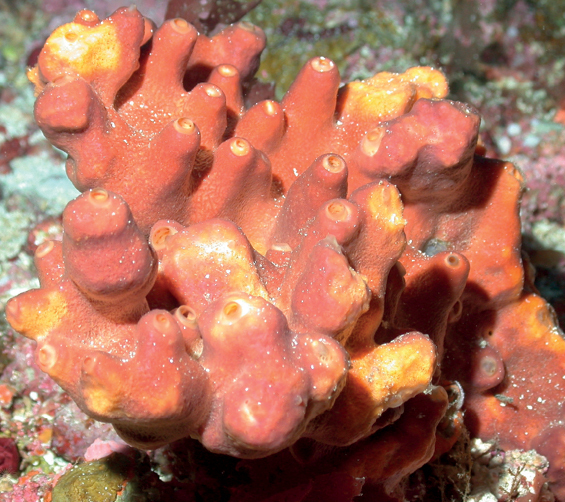
*Ectyoplasiaferox*, 60 m deep. Sample DFH9-12C.

**Figure 9.**
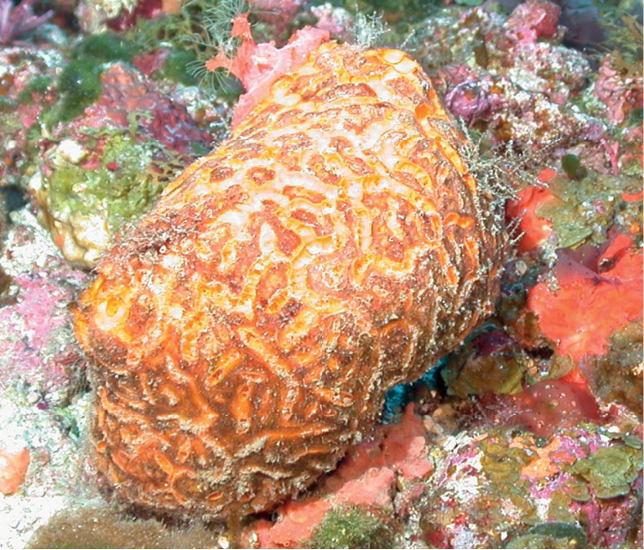
*Didiscusoxeatus*, 60 m deep. Sample DFH9-11B.

**Figure 10.**
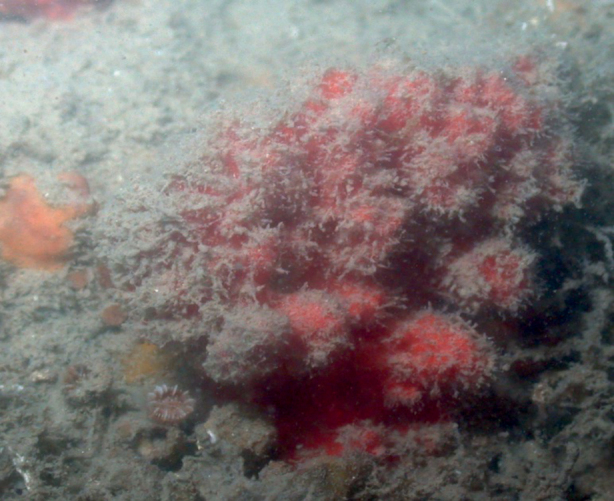
*Higginsiacoralloides*, specimen partially buried on sediment, 60 m deep. Note fine sediment on sponge. Samples DFH9-7A,7B.

**Figure 11.**
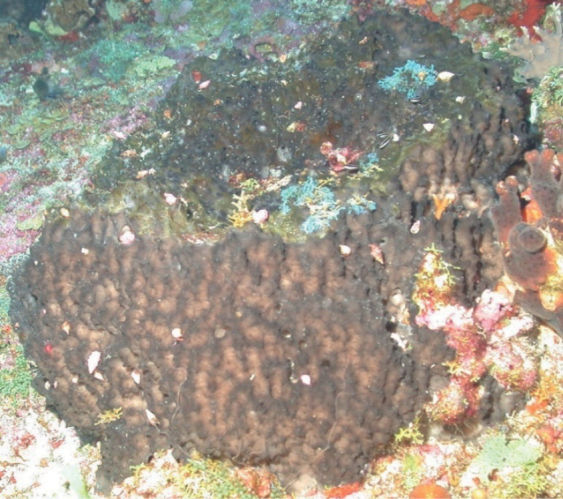
*Biemnacribaria*, 36 m deep. Photo code SP22.

**Figure 12.**
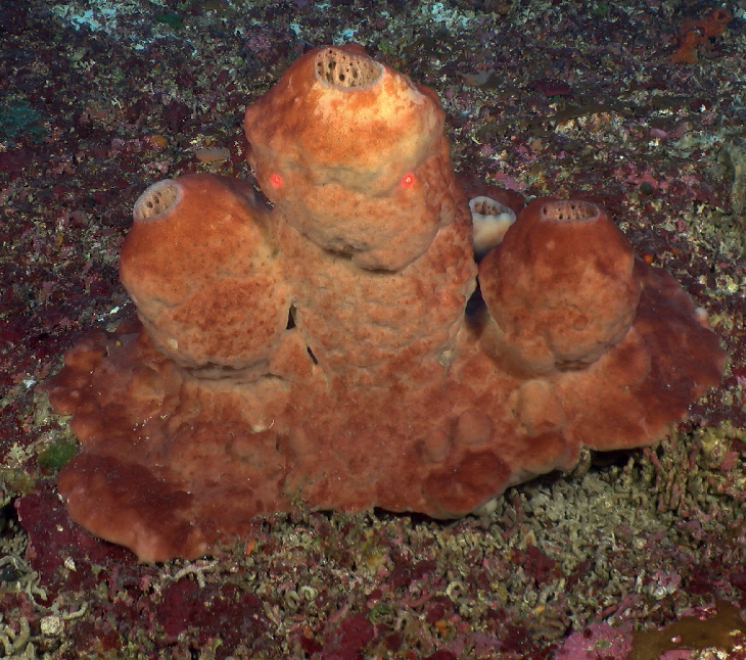
*Neofibularianolitangere*, 70 m deep. Sample GFOE3-30 (8-31-19).

**Figure 13.**
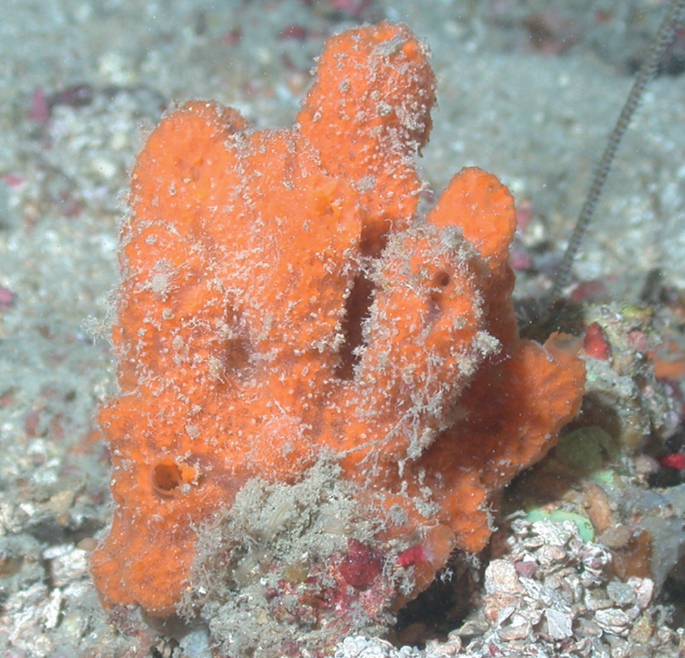
*Acanthellacubensis*, 68–88 m deep. Samples DFH9-14A DFH9-2A, DFH9-3D.

**Figure 14.**
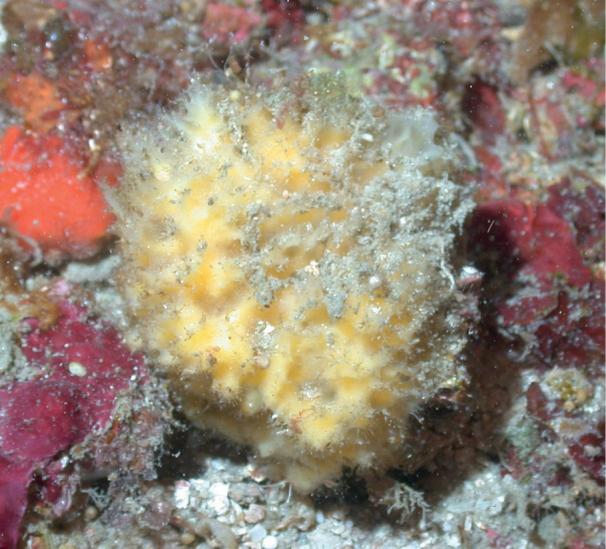
Acanthellacf.mastophora 80 m deep. Sample DFH9-13B.

**Figure 15.**
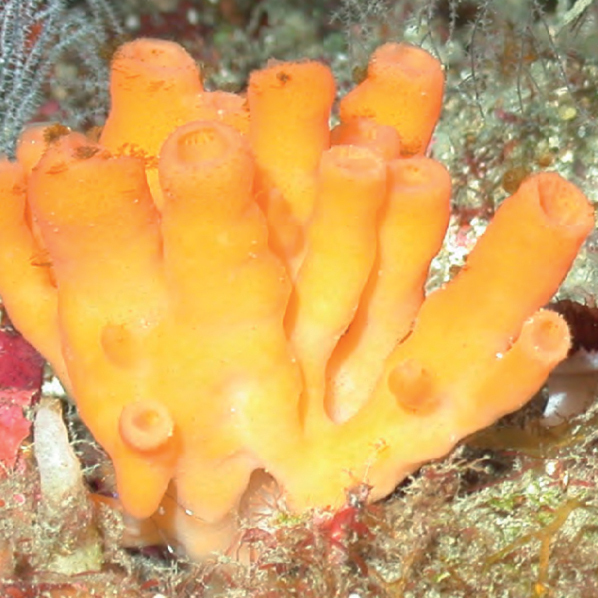
*Aulettatuberosa*, 72–78 m deep. Samples DFH9-13A, DFH9-4A.

**Figure 16.**
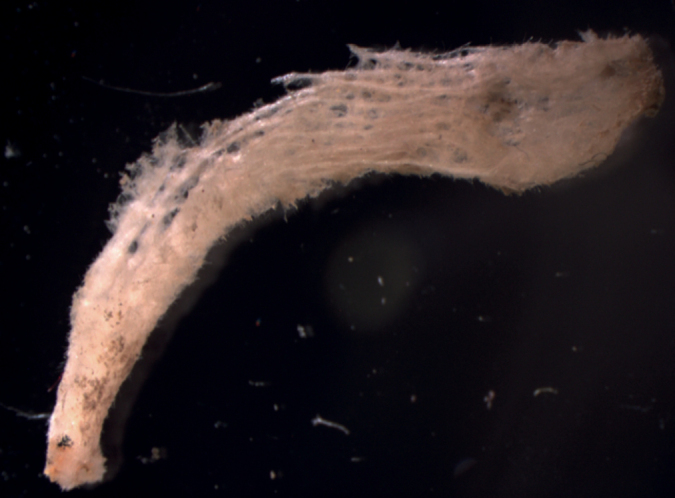
*Aulettasyncinularia*, young specimen, collected on a rock at 147 m deep. Sample GFOE3-23F.

**Figure 17.**
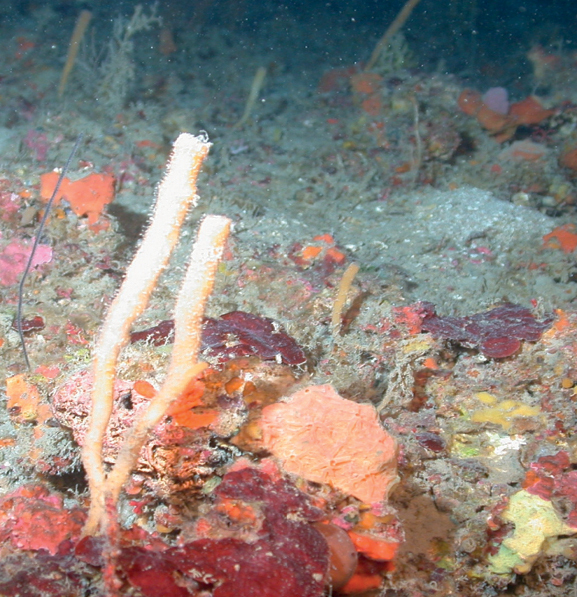
*Auletta*sp. nov. 1, 90–95 m deep. Samples DFH8-15A, GFOE 3-8H.

**Figure 18.**
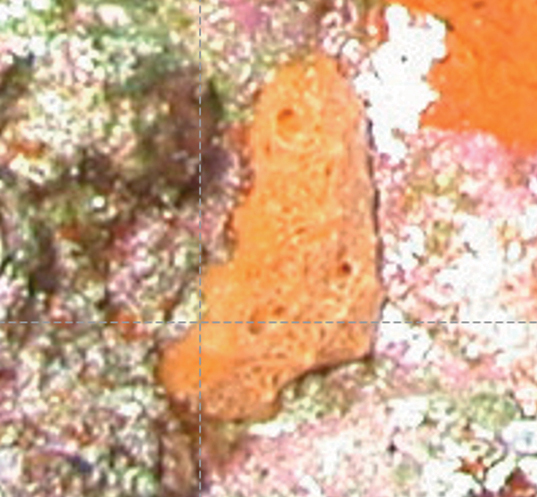
*Scopalinaruetzleri*, 50 m deep. Photo code SP23.

**Figure 19.**
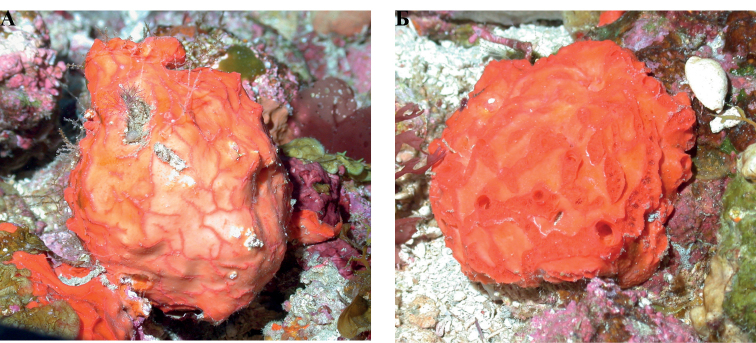
**A***Placospherastraantillensis*, with surface contracted, 60 m deep. Sample DFH9-10C **B***Placospherastraantillensis* relaxed, showing groves with ostia (incurrent pores), and oscula (excurrent openings), 60 m deep. Sample DFH9-11C.

**Figure 20.**
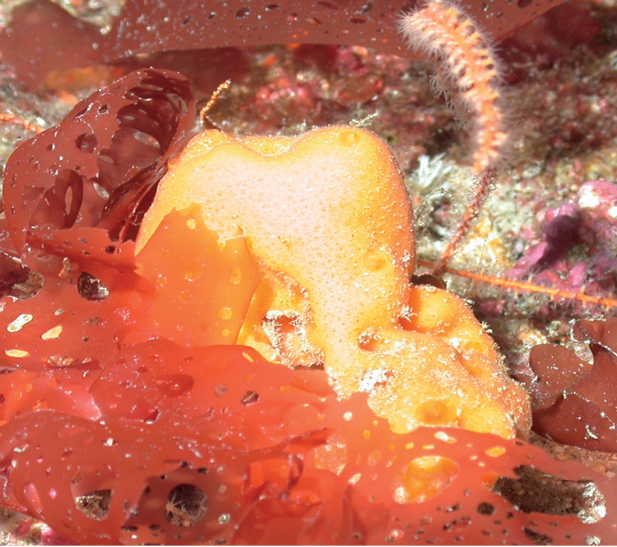
Haliclona (Reniera) sp. 1, 69 m deep. Sample DFH9-6E.

**Figure 21.**
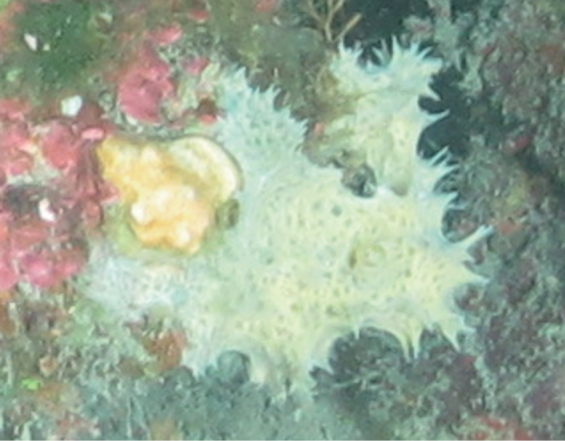
Callyspongiacf.armigera, 63 m deep. Photo code SP42. Sample DFH6-39-6.

**Figure 22.**
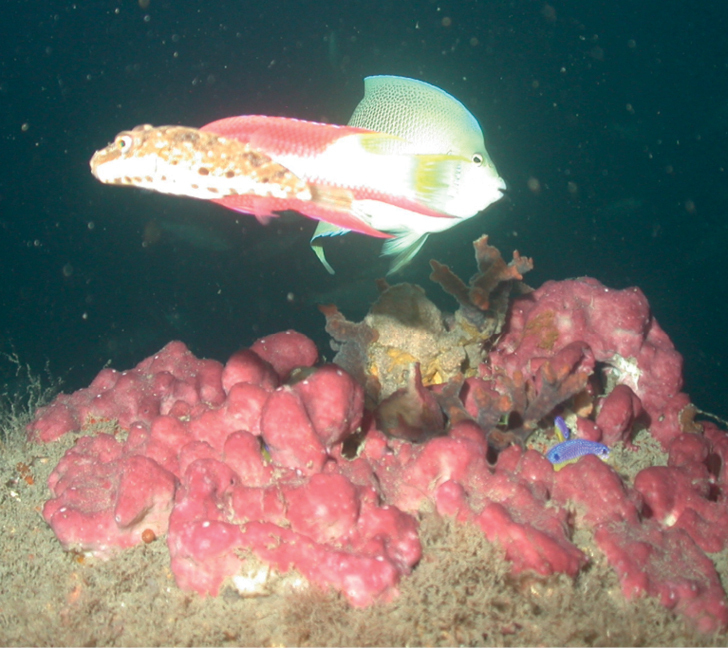
*Neopetrosiaproxima*, 53–60 m deep. Samples DFH8-37B, DFH9-7D, DFH9-9C.

**Figure 23.**
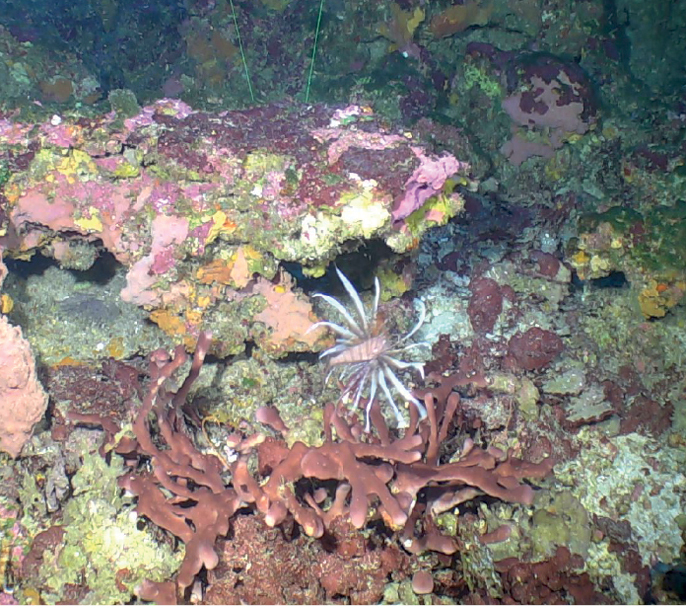
*Petrosia*sp. nov. 1, 73–79 m deep. Sample DFH33-542A.

Sixty-four species were identified from a collection of 54 samples with photographs and ten photographs (without samples) from inside and around Flower Garden Banks National Marine Sanctuary (Suppl. material 1), and 63 are synoptically described below (Figs 3–65). Two species belong to the class Hexactinellida (order Hexasterophora). Two species, Plakortiscf.simplex and *Plakinaversatilis* (not represented in the present guide, but a sample was studied by KR, SK, and CA) represent the class Homoscleromorpha (order Homosclerophorida), and 60 species represent the class Demospongiae (14 orders). The most diverse orders in terms of family diversity and species richness are: Tetractinellida (11 spp. within 5 families), Dictyoceratida (9 spp. within 4 families), Haplosclerida (10 spp. within 5 families), Axinellida (5 spp. within 4 families), and Bubarida (5 spp. within 1 family). The most species-rich genus with several undescribed species is *Ircinia* with five species distinguished. Forty-seven species are identified either to species level or with a probable intraspecific variation of a known species (12 spp. as cf.), or with affinity to a known species (one sp. as aff.). Fifteen species were given only a generic assignation; many of those probably represent undescribed species or require deeper taxonomic studies, such as museum type comparisons or molecular evaluation to confirm a species identification. One morphotype could only be identified to the family level: a skeletal-less member of the family Ianthellidae that thinly encrusted a portion of an Hexactinellida. If this taxonomic identification is correct, it would constitute the first association of this type ever reported.

### ﻿Geographic scope

Eleven species included in this study are either first reports for the occurrence of that species at mesophotic depths, or first occurrences in the Gulf of Mexico or specifically in the northwestern Gulf of Mexico. *Biemnacribaria*, *Placospherastraantillensis*, *Batzellarubra*, and *Erylustrisphaerus* are here reported at mesophotic depths (> 50 m deep) for the first time. First reports in the Gulf of Mexico include *Stellettinopsismegastylifera*, *Erylusalleni*, and *Erylusgoffrilleri*. First reports in the northwestern area of the Gulf of Mexico include *Agelasdilatata*, Neophrissospongiacf.nolitangere, *Erylustrisphaerus*, and *Irciniacampana*.

The occurrence and qualitative abundance estimate for most of the species in this study, along 17 banks or features in Flower Garden Banks National Marine Sanctuary and vicinity are summarized in the Suppl. material 1.

### ﻿Taxonomic accounts

**Phylum Porifera**

**Class Demospongiae**

**Subclass Heteroscleromorpha**

#### ﻿Order Agelasida

**Family Agelasidae**

##### ﻿ Agelas cf. citrina

Taxon classificationAnimaliaAgelasidaAgelasidae

Gotera & Alcolado, 1987

A5FDF094-5DFD-5923-8AA6-8294D9760C7E

Fig. 3

###### Diagnostic features.

Massive amorphous to thick crust (≤ 3 cm thick), bright orange to reddish externally. The surface is convoluted with irregular folds and depressions. Oscula round, sparse, 2–5 mm wide.

###### Similar species.

*Agelasclathrodes* has key-holed and round oscula. *Agelascerebriformis* has a convoluted surface but it is brown and tubular.

###### Distribution and abundance.

*Agelascitrina* occurs on shallow coral reefs throughout the Caribbean and the Florida Keys. Found on mesophotic reefs at FGBNMS, Cuba (50–61 m deep), and South Carolina MPA (52 m depth). At FGBNMS, rare to moderate in abundance and a widespread distribution occurring at 11 sites.

###### Ecology.

Lower mesophotic reefs and heavily silted reefs in FGBNMS region. A strong, particular garlic-like odor is associated with all regional variants. This morphotype is referred to as cf. since it does not have the typical flabellate shape nor the orangish color.

###### Identification.

KR, SK, CA, MCD.

###### References.

Díaz et al. 2019, 2021; Parra-Velandia et al. 2014; Zea et al. 2014.

##### ﻿ Agelas clathrodes

Taxon classificationAnimaliaAgelasidaAgelasidae

(Schmidt, 1870)

F9FA3972-5E78-52E0-A350-C19F7D185F0D

Fig. 4

###### Diagnostic features.

Massive, flabellate, orange reddish in color. The surface is rugose, irregularly riddled by round (1–10 mm wide) and key-holed (1–4 cm long) oscula.

###### Similar species.

*Agelascitrina* flabellate specimens are similar but lack key holed oscula and usually have a paler pinkish or yellowish color.

###### Distribution and abundance.

Common in shallow and mesophotic reefs in North and South Carolina, eastern Florida, throughout the Caribbean, the Guyana shelf, and Brazil.

###### Ecology.

Coral reefs, coral communities, and coralline algae reefs in FGBNMS region.

###### Identification.

MCD, MFN.

###### References.

Díaz et al. 2019; Parra-Velandia et al. 2014

##### ﻿ Agelas dilatata

Taxon classificationAnimaliaAgelasidaAgelasidae

(Duchassaing & Michelotti, 1864)

2C5654EA-7BE3-5CE4-8D70-E5DC6EA07204

Fig. 5

###### Diagnostic features.

Flabellate to fan- and cup-shaped, < 3 cm thick, sometimes pedunculated. Brown in color. The surface is smooth with abundant and homogeneously arranged round oscula (4–10 mm) on the upper side, and small unevenly dispersed ostia (1–2 mm wide) on the underside.

###### Similar species.

*Agelasdispar*, a fan-shaped brown species, which is thicker and possesses mostly key-holed oscula.

###### Distribution and abundance.

Previously considered restricted to the Bahamian-Greater Antilles shallow coral reefs (18–30 m deep) and Cuba (90–115 m). This is the first report for the NW GOM, where it is rare at Sonnier Bank.

###### Ecology.

Coralline algae reefs. Specimen is overgrown by a film of green algae. A unique alkaloid isolated from a Yucatan specimen is bioactive against a multidrug-resistant pathogen *Pseudomonasaeruginosa* (Pech-Puch et al. 2020).

###### Identification.

MCD.

###### References.

Díaz et al. 2019; Parra-Velandia et al. 2014.

#### ﻿Order Axinellida

**Family Axinellidae**

##### ﻿ Ptilocaulis cf. walpersii

Taxon classificationAnimaliaAxinellidaAxinellidae

(Duchassaing & Michelotti, 1864)

989D8FB6-C38D-5FDB-92C7-2A13BE09C5E0

Fig. 6

###### Diagnostic features.

Flagelliform branching; single or multiple branches (ca. 1–2 cm wide, ≤ 50 cm height). Red to orange in color. Branches have different lengths, and they can be straight, bent, or laterally fused forming flabellate bodies. Surface rugose and porous, with flattened or rounded processes. Oscula are sparse along the side of branches, hardly visible. Branches are compressible and firm. The identification given to this specimen is based on the external morphology and observations of the live photo.

###### Similar species.

*Ptilocaulismarquezi* (with oxeas and styles) and *Higginsiacoralloides* (with acanthose micro-oxeas added to large oxeas and styles). *Ptilocauliswalpersii* has only styles as spicules. The cf. is placed since the spicules could not be corroborated. *Higginsiacoralloides* consists of shorter (≤ 10 cm height) and thicker branches (3–5 cm wide).

###### Distribution and abundance.

*Ptilocauliswalpersii* is widely distributed on shallow coral reefs throughout the Caribbean, Florida, and Bermuda (0.5–35 m); recently reported at the southern GOM, 4–20 m deep (Ugalde et al. 2021). This is the first report from the northwestern GOM on mesophotic reefs. Common on Cuban mesophotic reefs. At FGBNMS, rare abundance and documented only at West Flower Garden Bank.

###### Ecology.

Coralline algae reefs.

###### Identification.

MCD, CA.

###### References.

Alvarez et al. 1998; Ugalde et al. 2021.

#### ﻿Order Axinellida

**Family Heteroxyidae**

##### ﻿ Myrmekioderma gyroderma

Taxon classificationAnimaliaAxinellidaHeteroxyidae

(Alcolado, 1984)

3EC78C05-D255-53D8-882A-7C881DCFE02A

Fig. 7

###### Diagnostic features.

Massive amorphous to thick encrusting (3.5 cm thick). Brown reddish to orange in color externally, orange internally. Surface highly ornamented by plates and shallow grooves. Oscula in low abundance and irregularly arranged.

###### Similar species.

Very similar to *Didiscusoxeatus*, and *Myrmekiodermarea*; this last species presents a smoother appearance and few thinner grooves. Only a spicule analysis allows to distinguish between them. *Didiscus*spp. have discorhabds as microscleres, *Myrmekiodermarea* has only straight trichodragmata, while *Myrmekiodermagyroderma* has twisted long trichodragmata.

###### Distribution and abundance.

Shallow and mesophotic reefs, throughout the Caribbean and Gulf of Mexico. At the FGBNMS the species presents low to medium abundance (2–100 individuals) at nine sites.

###### Ecology.

Coralline algae reefs, algal nodules, lower mesophotic reefs.

###### Identification.

KR, SK, CA, MCD.

###### References.

Alcolado 1984; Pomponi et al. 2019.

#### ﻿Order Axinellida

**Family Raspailiidae**

##### ﻿ Ectyoplasia ferox

Taxon classificationAnimaliaAxinellidaRaspailiidae

(Duchassaing & Michelotti, 1864)

911CF01B-FA7E-57C4-A0A4-49D538B55F03

Fig. 8

###### Diagnostic features.

Thickly encrusting to palmate. Brown to reddish externally, orange internally. Rugose to smooth surface. Oscula on tips of chimneys. Pale colored oscular membranes. Compressible, easy to break.

###### Similar species.

This species is quite variable in color and level of rugosity. Massive and smooth forms of *Clionavarians* can be confused with it. Spicule composition allows a definitive diagnosis.

###### Distribution and abundance.

Throughout the Caribbean, Gulf of Mexico, and SE Brazil, very common in shallow coral reefs. Mesophotic reefs in Cuba. At FGBNMS the species is rare to low in abundance (1–10) at five sites.

###### Ecology.

Coralline algae reefs, coral communities, algal nodules, lower mesophotic reefs.

###### Identification.

KR, SK, CA, MCD.

###### References.

Wiedenmayer 1977; Ugalde et al. 2021

##### ﻿ Didiscus oxeatus

Taxon classificationAnimaliaAxinellidaRaspailiidae

Hechtel, 1983

7F83B945-1274-54FB-9AC0-AF995B4F1036

Fig. 9

###### Diagnostic features.

Massive to crustose, brown reddish to orange in color externally, orange internally. Highly ornamented surface consisting of variously shaped plates and vermiform grooves. Few oscula, all with an orange membrane.

###### Similar species.

*Myrmekiodermagyroderma* and *Myrmekiodermarea* are very similar externally; the distinction of their microscleres allows their differentiation. *Didiscus*spp. have discorhabds and *Myrmekioderma*spp. have trichodragmata (see Boury Esnault and Rützler 1997.

###### Distribution and abundance.

Throughout the Caribbean, SE Brazil, and northern GOM on shallow reefs. Mesophotic reefs at FGBNMS, Lesser and Greater Antilles, Florida, Bahamas, and Brazil (Pomponi et al. 2019). At FGBNMS the species was found once at one site.

###### Ecology.

Coralline algae reefs, algal nodules.

###### Identification.

KR, SK, CA, MCD.

###### Reference.

Alcolado 1984.

#### ﻿Order Axinellida

**Family Stelligeridae**

##### ﻿ Higginsia coralloides

Taxon classificationAnimaliaAxinellidaStelligeridae

Higgin, 1877

049A36F2-5A05-5D0B-80D3-DF90FBB0D1AF

Fig. 10

###### Diagnostic features.

Bushy with several digitate branches diverging from a thicker peduncle. Vermillion red alive. The surface is composed of irregular tubercules, corrugations, or conules with projecting spicules that trap sediment; similar to a cauliflower surface, with interstitial areas where inconspicuous ostia and oscula can be found. Consistency is spongy but firm.

###### Similar species.

Younger specimens of *Ptilocaulismarquezi* (with oxeas and styles) and *Ptilocauliswalpersii* (with styles) might be confused with *Higginsiacoralloides* (with acanthose micro-oxeas added to large oxeas and styles).

###### Distribution and abundance.

Shallow coral reef and hard substrate at Guyana Shelf, Grenada, Bahamas, Florida, Nicaragua, Yucatan, North Carolina, possibly Brazil (van Soest 2017). Mesophotic depths at Brazil, Guyana, Eastern Antilles, Florida, and Bahamas, and northwestern GOM at FGBNMS. At FGBNMS it is rare to low (1–10) in abundance at six sites.

###### Ecology.

Lower mesophotic reefs, heavily silted reefs, coralline algae reefs.

###### Identification.

KR, SK, CA, MCD.

###### Reference.

Wiedenmayer 1977.

#### ﻿Order Biemnida

**Family Biemnidae**

##### ﻿ Biemna cribaria

Taxon classificationAnimaliaBiemnidaBiemnidae

(Alcolado & Gotera, 1986)

91A6F561-273E-5696-A3B8-73253877F7E2

Fig. 11

###### Diagnostic features.

Massive sub-spherical barrel growth form, with a top central dip. Color dark brown externally, tan internally. Multiple digitate projections on the surface on the top and side areas of the sponge. Oscula are aggregated on the concave upper depression.

###### Similar species.

The overall shape is reminiscent of other barrel sponges such as *Irciniastrobilina* or *Spheciospongiavesparium*, but the digitate projections of *Biemnacribaria*, and the skeleton allow their differentiation.

###### Distribution and abundance.

The sponge is rare in occurrence but reported at 20 m from Cuban and Jamaican reefs (Alcolado 1984; Lehnert and van Soest 1998). This is the first report at mesophotic depths and in the northwestern GOM at FGBNMS. At FGBNMS it is rare and was observed only once at Bright Bank.

###### Ecology.

Coral communities, coralline algae reefs.

###### Identification.

MCD.

###### Reference.

Lehnert and van Soest 1998.

##### ﻿ Neofibularia nolitangere

Taxon classificationAnimaliaBiemnidaBiemnidae

(Duchassaing & Michelotti, 1864)

41D2E3A3-2485-591D-978F-AF789D76E8A7

Fig. 12

###### Diagnostic features.

Massive base with thick lobes (10–30 cm high x 10–15 cm wide). Brown to yellowish in color externally, tan internally. The surface is irregularly corrugated to velvety smooth. The oscula are on top of lobes with a thin paler membrane. This species can grow as a thick barrel or massive crusts. The sponge is soft and friable in consistency. It is well known by its tendency to cause skin irritation.

###### Similar species.

The massive size and reddish-brown external color reminiscent of some *Neopetrosia*spp. Spicules allow clear differentiation.

###### Distribution and abundance.

Coral reefs or rock pavements in shallow depths in southwestern Caribbean (Colombia and Panama), Florida and North Carolina. At FGBNMS it is low to high (2–100+) in abundance at five sites. Thousands of polychaete worms swarmed from the inside of the sponge when it was collected.

###### Ecology.

Coralline algae reefs, algal nodules, and lower mesophotic reefs.

###### Identification.

KR, SK, CA, MCD.

###### Reference.

Wiedenmayer 1977.

#### ﻿Order Bubarida

**Family Dictyonellidae**

##### ﻿ Acanthella cubensis

Taxon classificationAnimaliaBubaridaDictyonellidae

(Alcolado, 1984)

6D2452E2-580E-552B-9398-595226A9ECE1

Fig. 13

###### Diagnostic features.

Massive digitiform to lobate with lobes 2 cm wide. Orange, spongy. Surface slightly rugose to conulose. Oscula 4–7 mm wide, on top of lobes, with transparent membranes. Soft, compressible.

###### Similar species.

*Ptilocauliswalpersii* (with only oxeas), *Ptilocaulismarquezi* (with oxeas and styles), while *Acanthellacubensis* has styles in a wide size range, and sinuous strongyles.

###### Distribution and abundance.

*Acanthellacubensis* occurs on shallow coral reefs in Cuba, south Caribbean, Florida, and North Carolina. At FGBNMS the species is from rare to medium (1–100) in abundance at 12 sites. The species occurs also at mesophotic rock pavements in South Carolina, inside proposed Charleston shelf MPA at 54 m (Díaz et al. 2021).

###### Ecology.

Coralline algae reefs, algal nodules, lower mesophotic reefs.

###### Identification.

CA, MCD.

###### Reference.

Alvarez et al. 1998.

##### ﻿ Acanthella cf. mastophora

Taxon classificationAnimaliaBubaridaDictyonellidae

(Schmidt, 1870)

BEC6F3EA-054F-5573-97AC-B3DA047AFA5F

Fig. 14

###### Diagnostic features.

Globular, slightly flattened (4 cm in diameter and 2–4 cm in height). Pale yellow. The surface has ‘woolly’-warty appearance due to roundish papillae. Between the papillae there are furrows (1–3 mm deep). The surface is very hairy due to abundant fibrous dense filaments of unknown origin, and projecting spicule brushes. Firm and compressible, with flexible surface projections. The cf. notation is due to predominance of oxea instead of styles. Otherwise, it is very similar to *Acanthellamastophora* in color, surface, and reticulate spicule arrangement.

###### Similar species.

Small specimens of *Cinachyrellaalloclada* may have similar appearance. Spicules would allow clear distinction.

###### Distribution and abundance.

*Acanthellamastophora* is found in south Florida, North Carolina, Azores and Eastern Atlantic (76–394 m deep). Widespread distribution at FGBNMS with rare to low (1–10) abundances at nine localities.

###### Ecology.

Coralline algae reefs, algal nodules, lower mesophotic reefs.

###### Identification.

MCD, CA.

###### Reference.

Alvarez et al. 1998.

##### ﻿ Auletta tuberosa

Taxon classificationAnimaliaBubaridaDictyonellidae

(Alvarez et al., 1998)

BEEAC328-9985-5111-BAD9-7C859CCCDAA7

Fig. 15

###### Diagnostic features.

Clusters of tubes (0.25 –1 cm diameter), arborescent, with short and narrow peduncle; tubes anastomose and are crooked, uneven, and bumpy. Orange to yellowish tan in color. The surface is felt-like, smooth visually. Oscula or vents, on top of the tubes (2–4 mm diameter). Soft and compressible in consistency.

###### Similar species.

the protuberances of the tubes and ramose thick branches make this a unique species. The spicules include slender oxeas, styles, and wavy strongyles allowing distinction from *Aulettasyncinularia*, which contains only styles and wavy strongyles.

###### Distribution and abundance.

*Aulettatuberosa* is reported from 60–80 m depth at Guyana, southern Caribbean, eastern Antilles, Florida, Bahamas, and southeast GOM (off Cape Sable) where it was originally described. At FGBNMS it has a wide-spread distribution, occurring at 12 sites, with abundance ranging from rare to common (1–100 individuals).

###### Ecology.

Coralline algae reefs, algal nodules, lower mesophotic reefs.

###### Identification.

MCD, CA.

###### Reference.

Alvarez et al. 1998.

##### ﻿ Auletta syncinularia

Taxon classificationAnimaliaBubaridaDictyonellidae

(Schmidt, 1870)

61FF629B-7E71-5081-B5A9-CF7E24582D9D

Fig. 16

###### Diagnostic features

**(young specimen).** Single white tube (1–3 mm wide, 1.6 cm high). Surface rugose. One oscule on top of the tube. As adults this species grows as a cluster of smooth slender tubes (3–10 cm long, 1 cm wide), with a peduncle (2–3 cm long). Spicules are highly conserved (sinuous strongyles and styles present in two or three size classes.

###### Similar species.

The young specimens of *Auletta*sp. nov. 1 (described below).

###### Distribution and abundance.

*Aulettasyncinularia* is reported from the Gulf of Mexico, Florida, Barbados, and Azores (70–159 m deep); elsewhere, down to 200 m depth (Alvarez et al. 1998). At FGBNMS the species was collected once at Elvers Bank growing on a rock with Hexactinellids and black corals.

###### Ecology.

This species was found associated with a rock where a large hexactinellid was growing (DFH3-23).

###### Identification.

MCD.

###### Reference.

Alvarez et al. 1998.

##### ﻿ Auletta

Taxon classificationAnimaliaBubaridaBubaridae

sp. nov. 1

AE76761A-B5E6-5246-8F4E-0C8E31D25B9A

Fig. 17

###### Diagnostic features.

Single or double slender tubes (0.5–1 cm in diameter), drab orange in color. Adult specimens are 8–13 cm long, and < 5 mm wide (DFH8-15A). A young specimen (GFOE 3-8H) was 2 cm high and 2–4 mm wide. Surface is smooth, microscopically hispid, and porous. Oscula on top of each tube are 4 mm wide. A very thin white membrane surrounds each oscule. Tubes are compressible, but they become harder and thinner towards the base.

###### Similar species.

The surface and slender tube shape of *Aulettasyncinularia* is similar to this undescribed species of *Auletta*. It differs from *Aulettasyncinularia* in having oxeas and anisoxeas (straight and sinuous) and lacking the size categories of styles.

###### Distribution and abundance.

Found at mesophotic reefs at FGBNMS. Wide-spread distribution in the FGBNMS with medium abundances, from low to common (2–100 individuals) at 13 sites.

###### Ecology.

Coralline algae reefs, algal nodules, lower mesophotic reefs.

###### Identification.

MCD, CA.

###### Reference.

Alvarez et al. 1998.

#### ﻿Order Scopalinida

**Family Scopalinidae**

##### ﻿ Scopalina ruetzleri

Taxon classificationAnimaliaScopalinidaScopalinidae

(Wiedenmayer, 1977)

D4787ABC-1BE8-528A-93BD-9D0A68503999

Fig. 18

###### Diagnostic features.

Thick encrusting, occasionally lobate, 1–2 cm thick. The color in life is bright orange to pinkish orange. The surface is conulose (1–2 mm high and 1–4 mm apart) with many large contractile ostia (500 µm wide). The oscula are 1–3 mm in diameter and have a delicate, transparent membrane. The consistency is very soft, delicate, limp, and easily tom.

###### Similar species.

Thicker specimens of *Prosuberiteslaughlini* (with tylostyles) may be confused in the field with *Scopalinaruetzleri* (with styles). Spicule analysis allows differentiation.

###### Distribution and abundance.

Common and widespread distribution in shallow water coral reefs and mangroves in the Caribbean, Bermuda, Brazil, and GOM. At FGBNMS it had low abundance (2–10) at two sites.

###### Ecology.

Coralline algae reefs, algal nodules.

###### Identification.

MCD.

###### Reference.

Wiedenmayer 1977.

#### ﻿Order Clionaida

**Family Clionaidae**

##### ﻿ Placospherastra antillensis

Taxon classificationAnimaliaClionaidaClionaidae

van Soest, 2009

A82B0E16-F276-5493-88AC-803B4F1506CB

Fig. 19

###### Diagnostic features.

Thick encrusting (1–5 mm thick). Color in life orange, dark orange, brown-orange, or yellowish. The surface consists of elongated plates, separated by meandering ridges and grooves with pores. The system of plates and ridges is irregular in shape. Consistency hard, rough to the touch.

###### Similar species.

The plates and canals on the surface are similar to *Placospongia*spp. surface. The intense orange color of *Placospherastraantillensis*, and the spicules allow their differentiation.

###### Distribution and abundance.

Usually under coral rubble and in reef caves, 20–23 m depth in Bonaire and Belize. First report at mesophotic depths. At FGBNMS the species has a widespread distribution occurring at 11 sites with rare to medium abundance (1–100).

###### Ecology.

Coralline algae reefs, algal nodules, lower mesophotic reefs.

###### Identification.

KR, SK, CA.

###### References.

van Soest, 2009; Rützler et al. 2014.

#### ﻿Order Haplosclerida

**Family Chalinidae**

##### ﻿ Haliclona

Taxon classificationAnimaliaHaploscleridaChalinidae

sp. 1

4C0D715F-6200-556C-980E-CC646DC0E22D

Fig. 20

###### Diagnostic features.

Massive encrusting (1 cm thick), orange in color. Surface smooth with tiny pores. Few oscula 2–3 mm wide. Compressible, soft, and crumbly.

###### Similar species.

This species can be confused with smooth specimens of *Pseudaxinellabelindae*, which is more red-orange in color and has styles for spicules instead of small oxea.

###### Distribution and abundance.

Mesophotic reefs in northwestern GOM at FGBNMS, and east GOM at Pulley Ridge (MCD, unpublished data). Rare to low abundance at two sites.

###### Ecology.

Coralline algae reefs, algal nodules, lower mesophotic reefs. This is probably an undescribed species of *Haliclona*.

###### Identification.

CA, MCD.

###### Reference.

de Weerdt 2000.

#### ﻿Order Haplosclerida

**Family Callyspongiidae**

##### ﻿ Callyspongia (Cladochalina) cf.armigera

Taxon classificationAnimaliaHaploscleridaCallyspongiidae

(Duchassaing & Michelotti, 1864)

F617034B-D6F7-5B58-AD1B-09D82B8E4E8A

Fig. 21

###### Diagnostic features.

Short repent branch, gray to cream in color, with thorny conules and a porous surface. Few oscula visible (3–4 mm wide). This species commonly grows as erect branches, although repent specimens are reported in the literature.

###### Similar species.

The abundant thorny conules and the stiff consistency of this species allows its morphological diferentiation, *Pleraplysilla* sp. 2 (Fig. 56) has similar pronounced but shorter conules and the sponge is quite soft and less porous that this species.

###### Distribution and abundance.

An occasional species in shallow coral reefs throughout the Caribbean, south GOM, and Florida. Found at mesophotic reefs in Cuba (52 m) and in northwestern GOM at FGBNMS, occurring in low abundance (2-10) at one site.

###### Ecology.

Coralline algae reefs.

###### Identification.

KR, SK, CA, MCD.

###### Reference.

Wiedenmayer 1977.

#### ﻿Order Haplosclerida

**Family Petrosiidae**

##### ﻿ Neopetrosia proxima

Taxon classificationAnimaliaHaploscleridaPetrosiidae

(Duchassaing & Michelotti, 1864)

2BA0014F-3D18-5524-9838-B6D0484C58C1

Fig. 22

###### Diagnostic features.

Thickly encrusting to massive lobate (2–9 cm in thickness). Pinkish to brown externally, tan internally. The surface is smooth but feels like sandpaper. Abundant oscula, 2–3 mm in diameter, and 1–3 cm apart. Oscula have a thin white membrane that contrasts with the darker surface color. The sponge is very firm to hard.

###### Similar species.

This species is similar to other species of *Neopetrosia* described by Vicente et al. (2019). Details of the surface and spicules allow differentiation.

###### Distribution and abundance.

A common species on shallow rocky shores and reefs to deeper reef habitats with a variety of wave exposures (Zea et al. 2014), also in caves (Perez et al. 2017). Found at mesophotic reefs on the northwestern GOM at FGBNMS and possibly in Cuba, identified as Petrosiidae CU-17 (Díaz et al. 2018). At FGBNMS the species has been found at three sites with abundances from rare to medium (1–100).

###### Ecology.

Coralline algae reefs, silted lower mesophotic reefs.

###### Identification.

KR, SK, CA, MCD.

###### Reference.

Vicente et al. 2019.

##### ﻿ Petrosia

Taxon classificationAnimaliaHaploscleridaPetrosiidae

sp. nov. 1

F392B40B-8DE9-5DD7-A8A6-425D5846E82E

Fig. 23

###### Diagnostic features.

Round to flattened branching (branches 1–2 cm wide), occasionally anastomosing, with roundish tips. Branches are erect, horizontal, or creeping along the substrate. Red-brown to purple externally and tan internally in live. The tips are paler in color. The surface is very smooth. White rimed oscula, 1–2 mm wide, separated by several cm. The sponge is compressible but firm.

###### Similar species.

The growth form of this Petrosia (Petrosia) species in unique.

###### Distribution and abundance.

Mesophotic reefs and rocky pavement in the northwestern GOM at FGBNMS, east GOM at Pulley Ridge, and in South Carolina (52–72 m) (Díaz et al. 2021). At FGBNMS the species presents rare to low (1–10) distribution at four sites.

###### Ecology.

Coralline algae reefs, algal nodules, lower mesophotic reefs. The purple color probably originates from endosymbiotic cyanobacteria *Synechococcusspongiarium*.

###### Identification.

MCD.

###### Reference.

van Soest 1980.

##### ﻿ Petrosia weinbergi

Taxon classificationAnimaliaHaploscleridaPetrosiidae

van Soest, 1980

728D341E-4330-5034-A9A8-ECE445998080

Fig. 24

###### Diagnostic features.

**Figure 24.**
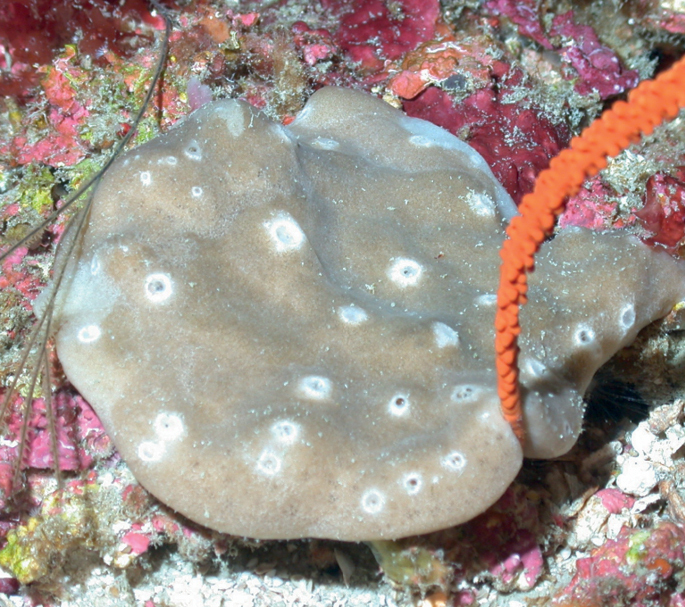
*Petrosiaweinbergi*, 69–71 m deep. Samples DFH9-3C, DFH9-6B.

Thick crusts (1–2 cm in thickness) to plate-shaped. Dark green to brown in color externally and tan internally, with oscula contrasting by a wide white rim. The surface is smooth to slightly undulating. The oscula are slightly raised from the surface and white, 1–2 mm wide and 2–5 cm apart. Usually, this species forms small patches, and the specimen in Fig. 24 is 8 × 6 × 1.5–2 cm. The sponge is hard, barely compressible.

###### Similar species.

The ear-shaped specimens of *Petrosiaweinbergi* are similar to *Petrosiapellasarca*. The former is greenish in color and lacks the small toxa. Similar species: include crustose forms of *Clionavarians* and *Clionaaprica* may look similar to crustose forms of *Petrosiaweinbergi*.

###### Distribution and abundance.

This species is rare in shallow reefs throughout the Caribbean and at mesophotic reefs in the Greater Antilles, Guyana, Brazil and in the northwestern GOM at FGBNMS. At FGBNMS the species is found in rare to low (1–10) abundance at two localities. Depth ranges from 8–500 m.

###### Ecology.

Coralline algae reef, algal nodule, lower mesophotic reef.

###### Identification.

KR, SK, CA, MCD.

###### References.

Pomponi et al. 2019; van Soest 1980, 2017.

##### ﻿ Xestospongia muta

Taxon classificationAnimaliaHaploscleridaPetrosiidae

(Schmidt, 1870)

D8B90636-79AA-592D-87C8-4DB3376CE276

Fig. 25

###### Diagnostic features.

Barrel-shaped. with a wide apical vent surrounded by a 2–5-cm wall which thickens towards the sponge base. Smaller specimens may present as a cone-shaped form. Red-brown externally, tan internally. Surface ranges from smooth to irregularly ridged or pitted. Few small openings (2–3 mm in diameter) may be oscula. Inner wall without detachable dermis, rough. The detachable dermis ends on the inside rim of the vent. The atrial cavity extends to ca. half the cup height. Consistency is brittle, easily crumbled.

**Figure 25.**
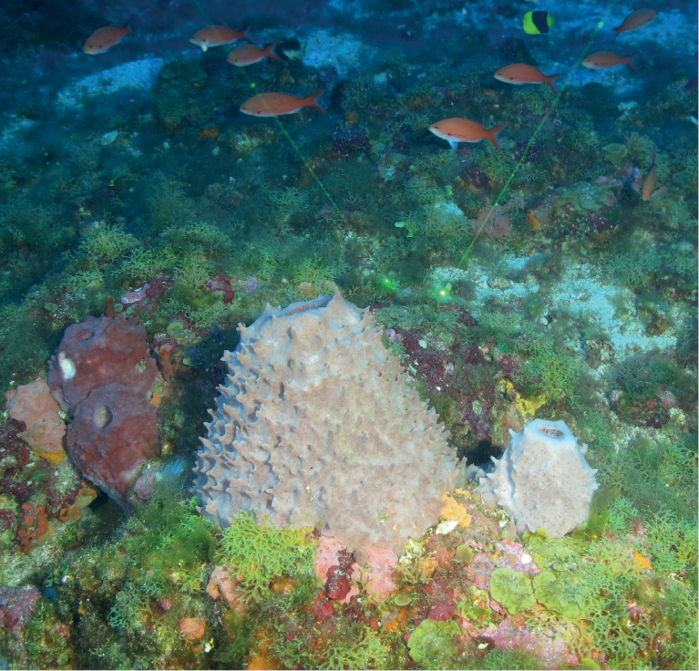
*Xestospongiamuta* (2 specimens on the right) and *Xestospongia*sp. nov. 1 (2 roundish dark red specimens on the left) at McGrail Bank, 58 m. Photo 202205114-T-161120_0004 (HBOI-FAU 05-2022).

###### Similar species.

*Xestospongia*sp. nov. 1, described below, is shorter, with a flat top, thicker rimmed walls, and much smaller atrium than *X.muta*.

###### Distribution and abundance.

An iconic species from shallow reefs in Florida, throughout the Caribbean, to southeastern Brazil, southern GOM, and northwestern GOM at FGBNMS. Mesophotic reefs in Cuba, south Florida, northwestern GOM at FGBNMS, and east GOM at Pulley Ridge. Reed (2022) reports it at three banks in FGBNMS (51–69 m deep).

###### Ecology.

Coral reefs, coral communities, coralline algae reefs, algal nodules.

###### Identification.

John Reed, MCD.

###### Reference.

Díaz et al. 2019; van Soest 1980.

##### ﻿ Xestospongia

Taxon classificationAnimaliaHaploscleridaPetrosiidae

sp. nov. 1

4547C9AB-6554-5A66-A033-D6B9A385AEC8

Fig. 26

###### Diagnostic features.

Massive thick barrel sponge, with rounded edges and a small apical oscule or pseudo-oscule (3 cm in diameter). The sponge top is flattened. The color is pink to dark reddish, with whitish spots, tan internally. The surface is smooth to spikey and microscopically porous. A very thin transparent membrane can be distinguished on the oscule rim, and branching thin spicule tracts can be distinguished at high magnification.

**Figure 26.**
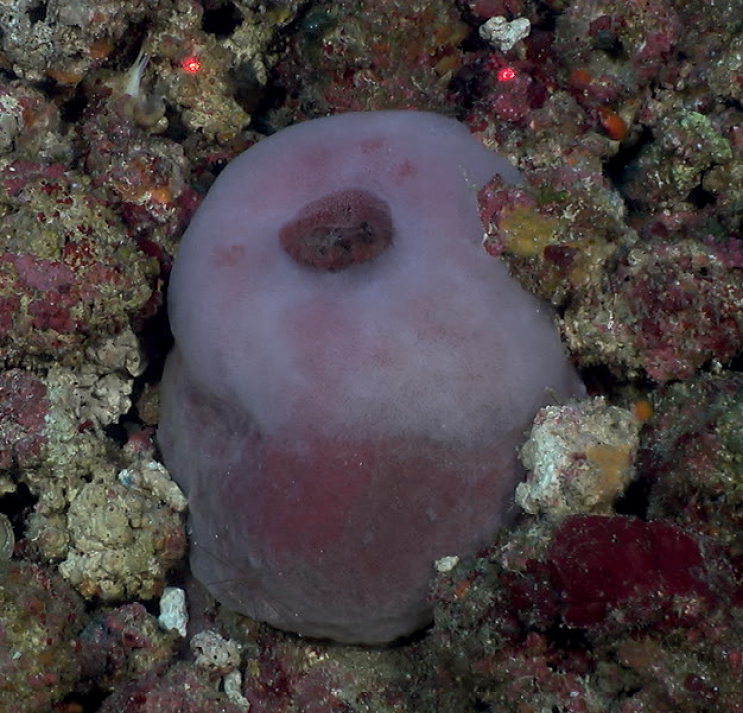
*Xestospongia*sp. nov. 1, 56 m deep. Sample GFOE3-27.

###### Similar species.

This species is similar to *Xestospongiamuta* but it is fat, shorter, with a flat top, thicker walls, and a smaller “atrium” than *Xestospongiamuta*.

###### Distribution and abundance.

Mesophotic reefs in Cuba, south Florida, northwestern GOM at FGBNMS region, and east GOM at Pulley Ridge. This was the most abundant species at the mesophotic reefs in Cuba, and it is currently being described by a Cuba-USA team. At FGBNMS, it has been recognized once at Geyer Bank; probably confused with *Xestospongiamuta* previously.

###### Ecology.

Algal nodules.

###### Identification.

KR, SK, CA, MCD.

###### Reference.

Díaz et al. 2019.

#### ﻿Order Haplosclerida

**Family Niphatiidae**

##### ﻿ Niphates erecta

Taxon classificationAnimaliaHaploscleridaNiphatiidae

Duchassaing & Michelotti, 1864

CB557425-888D-5BCF-86D5-BA4AC50AA118

Fig. 27

###### Diagnostic features.

Single erect branch to multiple branches or arborescent. Pink to gray in color. The surface is porous, microhispid, and rough to the touch. Many oscula dispersed along the branch with a slight elevation compared to the surface. Many oscula had a barnacle inside. The sponge is firm, slightly compressible.

**Figure 27.**
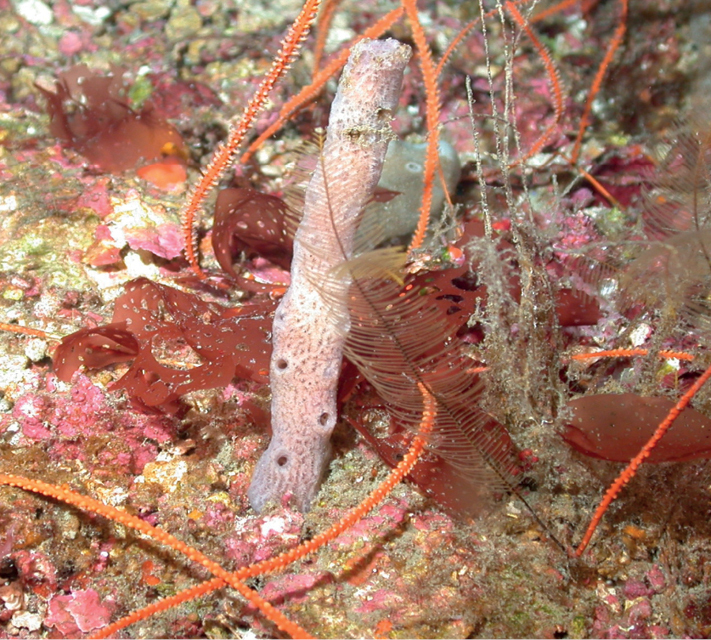
*Niphateserecta*, 71 m deep. Sample DFH9-6A.

###### Similar species.

*Niphatesamorpha* with erect branches and *Niphateserecta* can be confused. The possible conspecificity of these two forms remains to be clarified.

###### Distribution and abundance.

Very common species throughout the Caribbean, Bermuda, Florida, and Brazil at shallow (< 50 m) and mesophotic depths (50–100 m). At FGBNMS the species is reported with rare to high abundance (1–100+) at seven localities.

###### Ecology.

Coralline algae reef, algal nodule, lower mesophotic reef.

###### Identification.

KR, SK, CA, MCD.

###### References.

van Soest 1980. Pomponi et al. 2019.

#### ﻿Order Haplosclerida

**Family Phloeodictyidae**

##### ﻿ Siphonodictyon

Taxon classificationAnimaliaHaploscleridaPhloeodictyidae

sp. nov. 1

C3A6249B-9CB1-5154-A034-6552C9BAF566

Fig. 28

###### Diagnostic features.

Large massive sponge with abundant long yellow brown oscular tubes (3–12 cm long and 1.5–2 cm wide) that project between shorter, amorphous to digitate drab yellow fistules (1–4 cm high and < 10 cm long). Only a soft and smooth oscular tube was collected and had only oxeas as spicules. 18S sequences (738 bp) show that this species is separated phylogenetically from a clade formed by sequences of *Siphonodictyoncoralliophagum* and *Siphonodictyonbrevitubulatum* available on GenBank (Diaz, Segura, and Pomponi, unpublished data).

**Figure 28.**
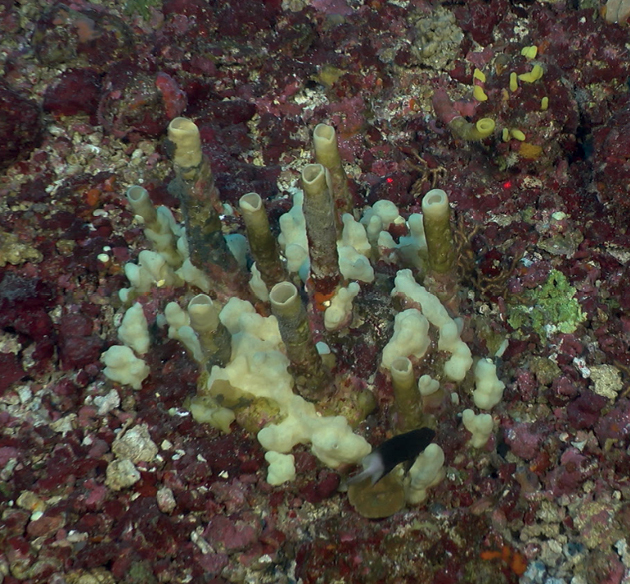
*Siphonodictyon*sp. nov. 1, 67 m deep. Sample GFOE3-2.

###### Similar species.

*Oceanapia*spp. may have similar oscular tubes and fistules (Santos Neto et al. 2018). The genetic data was essential to provide the generic assignation to this species.

###### Distribution and abundance.

The species was seen once at Geyer Bank at FGBNMS.

###### Ecology.

Found in algal nodule beds. This species is a bio-eroding sponge.

###### Identification.

Iris Segura, KR, MCD.

###### Reference.

Ruetzler 1971; Ruetzler et al. 2014.

##### ﻿ Siphonodictyon brevitubulatum

Taxon classificationAnimaliaHaploscleridaPhloeodictyidae

Pang, 1973

53BBB52A-082D-5A69-880F-4D3537A70541

Fig. 29

###### Diagnostic features.

Small abundant fistules (0.5–1 cm wide, 1–3 cm high) and rare long oscular tubes (1.5 cm in diameter), bright yellow in color. Smooth surface of fistules and oscular tubes.

**Figure 29.**
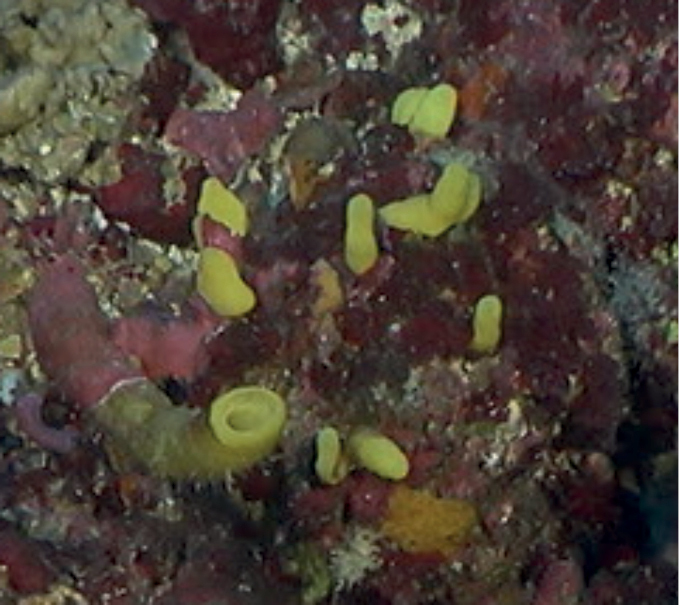
*Siphonodictyonbrevitubulatum*, 67 m deep. Inset taken from Fig. 28 upper right.

###### Similar species.

*Siphonodictyoncoralliophagum* has much larger and thicker bright yellow oscular tubes and fistules.

###### Distribution and abundance.

The species is reported from Jamaica, Costa Rica, Colombia, and Martinique, and northwestern GOM in the FGBNMS. At the FGBNMS it was observed once at Geyer Bank, while analyzing the photograph of *Syphonodictyon*sp. nov. 1 (Fig. 28). This is the first report of this species at mesophotic depths.

###### Ecology.

Algal nodules. This species is a bio-eroding sponge.

###### Identification.

MCD.

###### Reference.

Pang 1973.

#### ﻿Order Poecilosclerida

**Family Chondropsidae**

##### ﻿ Batzella rubra

Taxon classificationAnimaliaPoeciloscleridaChondropsidae

(Alcolado, 1984)

DD7F025A-9B59-5C0A-B32F-80C8D01A735E

Fig. 30

###### Diagnostic features.

Thinly encrusting sponge, growing over dead coral or other sponges. Deep orange to bright red color in live. The surface is smooth and ornamented by paler colored dermal canals that branch away from the oscula, wide close to the oscula and thinner away from it. The consistency is compressible where the sponge is thicker.

**Figure 30.**
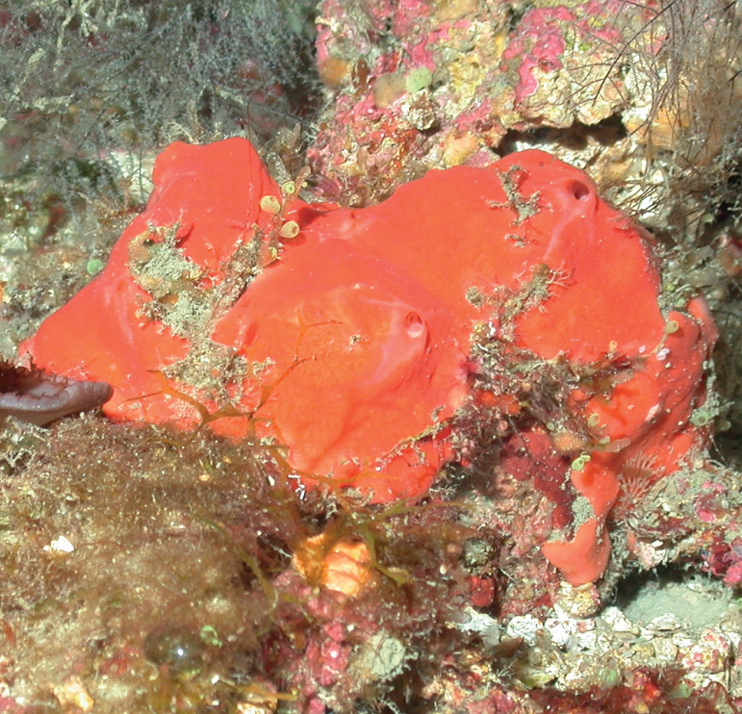
*Batzellarubra*, 90 m deep. Specimen observed on the photo of sample DFH9-13A.

###### Similar species.

The sponge can be confused with other red encrusting species, but the particular ‘dripping’ morphology of the dermal canals makes them easy to distinguish.

###### Distribution and abundance.

This species is reported from shallow reefs in Cuba and Bahamas. This is the first report for the species at mesophotic reefs. At FGBNMS the specimen on the photograph was found at east Flower Garden Bank and the species has rare to moderate abundance at ten other sites.

###### Ecology.

Coralline algae reefs, algal nodules, lower mesophotic reefs.

###### Identification.

MCD.

###### Reference.

Alcolado 1984; Zea et al. 2014.

##### ﻿ Batzella cf. rubra

Taxon classificationAnimaliaPoeciloscleridaChondropsidae

(Alcolado, 1984)

406596E4-DF0B-510C-97DC-908D934C9F03

Fig. 31

###### Diagnostic features.

Thinly encrusting sponge (1–10 mm thick) growing over dead coral. Orange to red color in life, black to purple in alcohol. The surface is smooth to bumpy with whitish branching dermal canals, and roundish papillae with two or three clumps of ostia. The cf. is due to the rounded papillae only described for *Batzellamollis*, a species found at the “Juan Fernandez and Desventuradas islands” off the Chilean Pacific coast.

**Figure 31.**
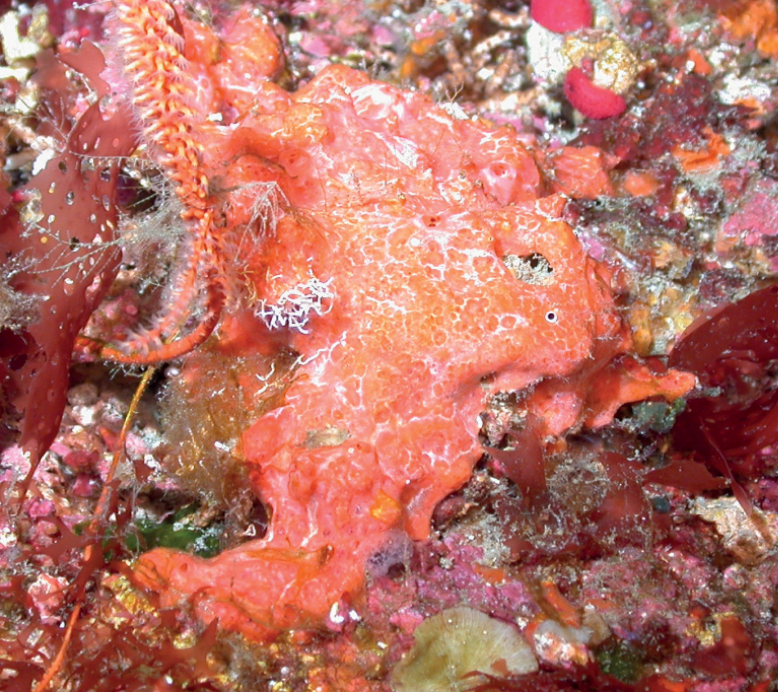
Batzellacf.rubra, 70 m deep. Sample DFH9-6F.

###### Similar species.

Esteves et al. (2018) describe three tropical western Atlantic *Batzella*spp: *Batzellarubra* (deep orange-red, smooth), *Batzellaficus* (dark brown), and *Batzellacataniresis* (yellow). *Monanchoraarbuscula* in its orange morphotype can be confused with Batzellacf.rubra.

###### Distribution and abundance.

This is the first record from mesophotic reefs. At FGBNMS the species has been recorded with rare to low abundance (1–10) in three localities.

###### Ecology.

Coralline algae reefs, algal nodules, lower mesophotic reefs.

###### Identification.

KR, SK, CA, MCD.

###### References.

Alcolado 1984; Esteves et al. 2018.

#### ﻿Order Poecilosclerida

**Family Microcionidae**

##### ﻿ Clathria

Taxon classificationAnimaliaPoeciloscleridaMicrocionidae

sp. 1

44071E3B-7257-5C2D-9CEA-575F8E964B59

Fig. 32

###### Diagnostic features.

Massive with short, protruding, flattened, digitate branches, forming a roundish bush. Colored red in life. Surface minutely porous and with a translucent veil (dermis). Oscula not visible. This species belongs to the genus *Clathria*; however, species identifications require spicule analysis.

**Figure 32.**
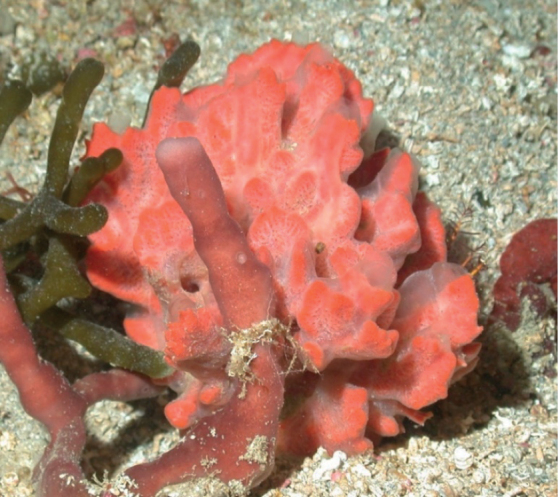
*Clathria* sp. 1, 63 m deep. Photo code SP03.

###### Similar species.

Several branching bushy *Clathria* species are described by Gomez (2014). Few of those species have been photographed alive, and their appearance changes dramatically once they are taken out of the water.

###### Distribution and abundance.

Arborescent *Clathria* species are diverse and well-known from the Gulf of Mexico (Gomez 2014). At the FGBNMS this species has been documented on mesophotic depths at three sites.

###### Ecology.

Sandy substrates.

###### Identification.

KR, SK, CA, MCD.

###### Reference.

Gomez 2014.

#### ﻿Order Suberitida

**Family Halichondriidae**

##### ﻿ Halichondria

Taxon classificationAnimaliaSuberitidaHalichondriidae

sp. 1

E917EA9A-2CA3-549F-8215-D191992CD878

Fig. 33

###### Diagnostic features.

Massive to thick encrusting, or globular to lobate with lobes ≤ 15 cm high. Yellow orange sponge alive, tan pinkish in alcohol. The surface is rugose to verrucose, with deep grooves and holes < 0.5 mm wide; the deep grooves, where thin ectosome is absent, have a feathery appearance. Oscula 0.5–2 cm wide, with a yellow membranous collar that is 5–8 mm high when the oscula are fully open. Compressible in consistency.

**Figure 33.**
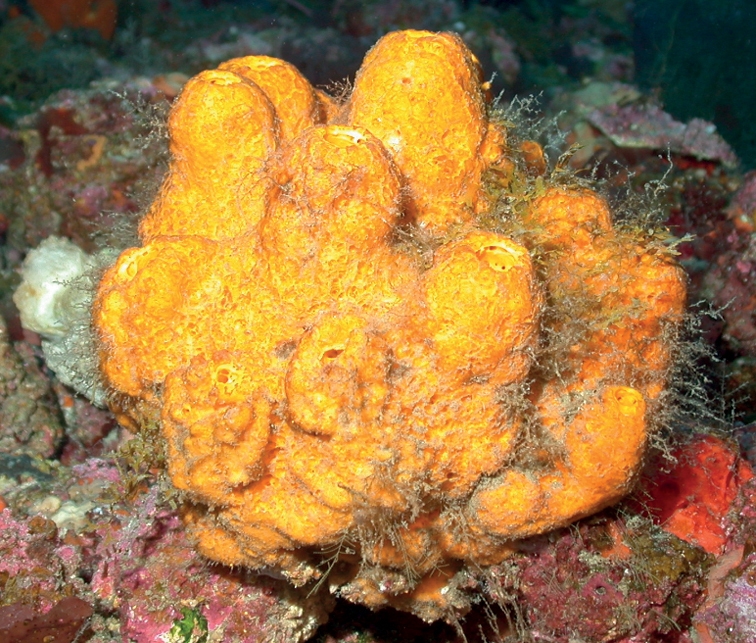
*Halichondria* sp. 1, 60–80 m deep. Samples DFH9-11D, DFH9-12D, DFH9-14E, DFH9-3E.

###### Similar species.

*Myrmekiodermarea* when it grows as a thick massive crust, and massive lobate *Axynissaambrosia* can be easily confused with this species. Its spicules (oxea in a wide size range) and surface features are unique among the Halichondriidae.

###### Distribution and abundance.

The sponge is common at FGBNMS on mesophotic habitats between 55–73 m. This is an undescribed species. The species occurs at four sites with rare to medium abundance (1–100).

###### Ecology.

Coralline algae reefs, algal nodules, lower mesophotic reefs.

###### Identification.

CA, SWK, MCD.

###### References.

Díaz et al. 1993; Zea et al. 2014.

##### ﻿ Topsentia bahamensis

Taxon classificationAnimaliaSuberitidaHalichondriidae

Díaz, van Soest & Pomponi, 1993

BE2358C2-9274-5628-8654-0806FBBE998D

Fig. 34

###### Diagnostic features.

Massive, columnar shape with round or blunt tip (10 cm high, 2–4 cm in diameter). The sponge is red-brown externally and tan internally in living sponges. The surface is smooth visually with a sandpaper feel. Five dispersed oscula (1–3 mm in diameter). Very firm in consistency, but crumbly.

**Figure 34.**
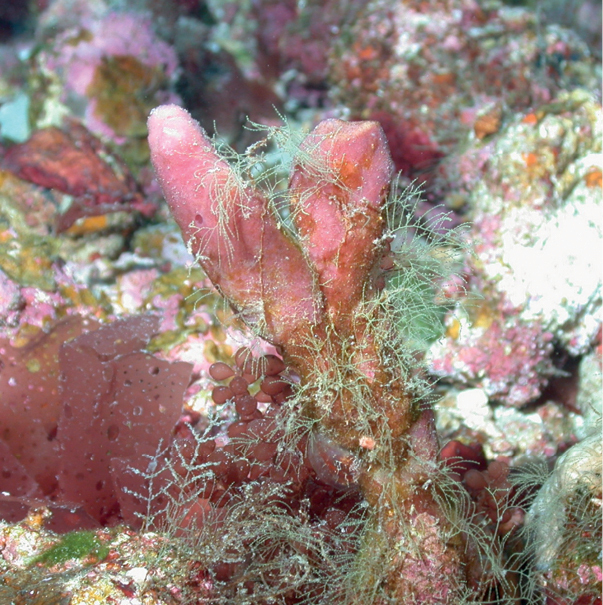
*Topsentiabahamensis*, 60 m deep. Sample DFH9-11F.

###### Similar species.

*Topsentiaophirhaphidites*, which has deformed small oxea added to the larger oxea. Petrosiids in general by their reddish brown color and hard brittle consistency. Spicule study needed to distinguish it.

###### Distribution and abundance.

Currently reported from shallow reefs in southern GOM in northern Yucatan and Belize, and at mesophotic depths in the Bahamas and northwestern GOM at the FGBNMS.

###### Ecology.

Coralline algae reefs, algal nodules.

###### Identification.

CA, SWK, MCD.

###### Reference.

Díaz et al. (1993).

#### ﻿Order Suberitida

**Family Suberitidae**

##### ﻿ Rhizaxinella clava

Taxon classificationAnimaliaSuberitidaSuberitidae

(Schmidt, 1870)

901A8312-621D-5079-8A3E-98056BB7A961

Fig. 35

###### Diagnostic features.

The “corn dog sponge”. A pale brown, clavate, stipitate sponge (15 cm in total length) with a long thin peduncle (2 mm in diameter at attachment area) and an upper globose soft body (1 cm in diameter at its thickest part). The surface is very smooth and velvety. The apical oscule is slit-shaped and has a collared membrane visible in the *in-situ* photograph. The sponge is firm but slightly compressible.

**Figure 35.**
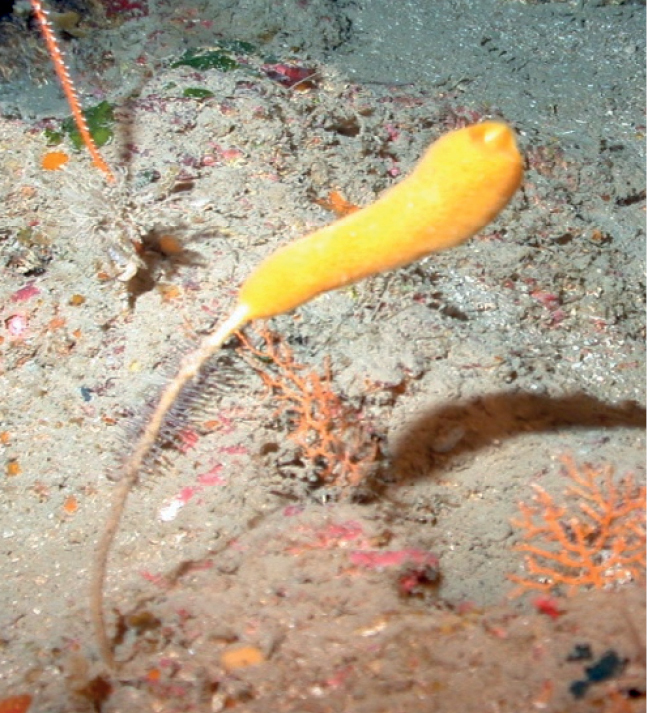
*Rhizaxinellaclava*, 106 m deep. Samples DFH6-42-4, DFH8-5A.

###### Similar species.

The “corn dog sponge” is similar externally to the “lollipop sponge”, *Stylocordylachupachups* and to other members of the family Stylocordylidae, which are mostly present in cold deep waters.

###### Distribution and abundance.

Currently reported at mesophotic depths in the Florida Keys, Guyana, Surinam, and northwestern GOM at the FGBNMS. At FGBNMS the species is widespread, occurring at 15 sites with abundance from rare to very common (1–100+).

###### Ecology.

Coralline algae reefs, silted lower mesophotic reefs.

###### Identification.

KR, CA, SWK, MCD.

###### Reference.

Pomponi et al. 2019; van Soest 2017.

#### ﻿Order Tetractinellida

**Sub-order Astrophorina**

**Family Corallistidae**

##### ﻿ Corallistes typus

Taxon classificationAnimaliaTetractinellidaCorallistidae

(Schmidt, 1870)

06D99CA1-8B51-5CFE-9E41-1FD49498E401

Fig. 36

###### Diagnostic features.

Small cups or plates, with undulating rims, walls 1–3 cm thick, usually with a thick peduncle. Brown with faintly pink tinges. The surface is smooth, with rims sometimes covered by sediment. Oscula not visible.

**Figure 36.**
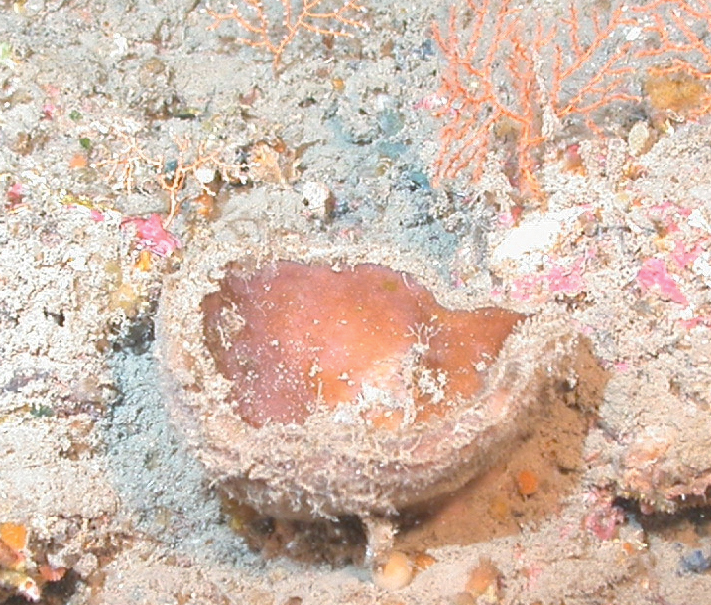
*In-situ* photo of *Corallistestypus*, 60–108 m deep. Samples GFOE3-23G, DFH8-10B.

###### Similar species.

An integrative study of 247 “lithistid” samples from the tropical western Atlantic (Schuster et al. 2021) encounters possibly six different undescribed species of *Corallistes*, similar to *C.typus*. Species of the genus *Neophrissospongia* have a similar appearance to this species.

###### Distribution and abundance.

Southern, eastern, and northern Caribbean, Florida, and Bahamas 61–914 m deep (Pomponi et al. 2001). Abundances increase from 150 m to 900 m deep. At FGBNMS the species is widespread at 17 sites with low to medium abundance (2–100).

###### Ecology.

Coralline algae reefs, lower mesophotic reefs.

###### Ideentification.

KR, CA, SWK.

###### References.

van Soest and Stentoft 1988; Schuster et al. 2021.

##### ﻿ Neophrissospongia cf. nolitangere

Taxon classificationAnimaliaTetractinellidaCorallistidae

Schmidt, 1870

ECBD96F6-36BB-5310-A1C2-14348C1F77F6

Fig. 37

###### Diagnostic features.

Plate or ear-shaped sponges with 1–2 cm thick walls with rounded margins, and 8–12 cm across. Tan-brown, plate. The cf. denomination was given since minute oscula on inner surface (0.5–1 mm wide) and a pedicel described for *Neophrissospongianolitangere* were not seen in the image or during voucher analysis.

**Figure 37.**
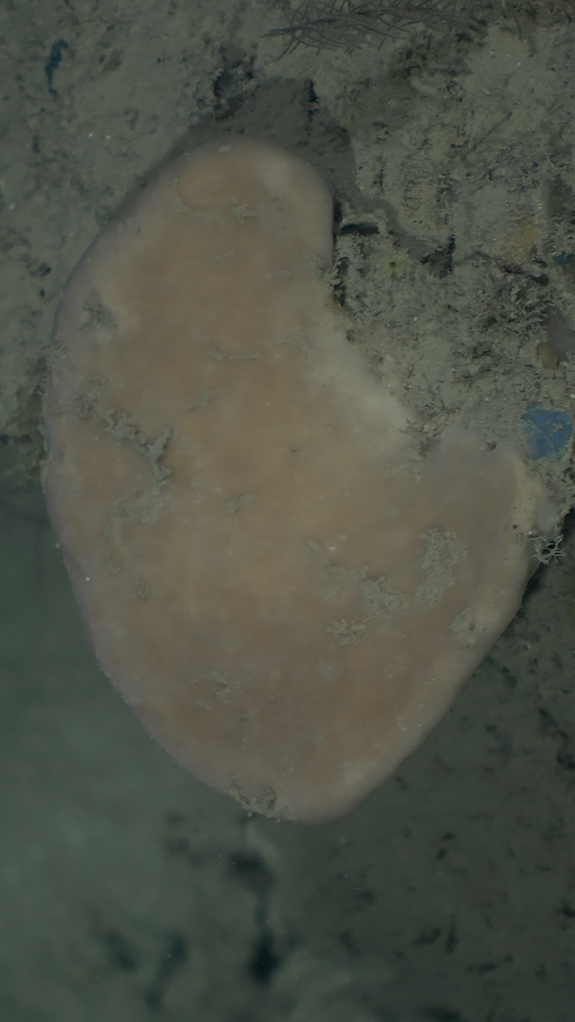
Neophrissospongiacf.nolitangere, 145 m deep. Sample GFOE3-1.

###### Similar species.

*Corallistestypus* and other species from the same genus. *Neophrissospongiadiffers* from *Corallistes* by the spiny or tuberculate nature of the dichotriaene top in the former, and the smooth nature on the later.

###### Distribution and abundance.

*Neophrissospongianolitangere* is common at deep waters from the eastern Atlantic, Azores and Mediterranean. Schuster et al. (2021) report at least 4 undescribed species of this genus in the tropical western Atlantic. This is the first report of the genus for the northwestern GOM, at FGBNMS seen once at one site.

###### Ecology.

Algal nodules.

###### Identification.

KR, CA, SWK, MCD.

###### References.

Pissera and Levi 2002; Schuster et al. 2021

#### ﻿Order Tetractinellida

**Sub-order Astrophorina**

**Family Ancorinidae**

##### ﻿ Stellettinopsis cf. megastylifera

Taxon classificationAnimaliaTetractinellidaAncorinidae

(Wintermann-Kilian & Kilian, 1984)

2E5DACDB-AB95-5172-A69F-27A7A8CC1CB3

Fig. 38

###### Diagnostic features.

Round to massive sponge. Brownish gray to dirty white in color. The surface is prickly, hispid, feels like sandpaper; numerous holes ≤ 3–5 mm in diameter in sponge body. Few larger oscula 1–4 cm wide. Hard and dense in consistency. Species identity requires further analysis and comparative work (Sandes et al. 2020).

**Figure 38.**
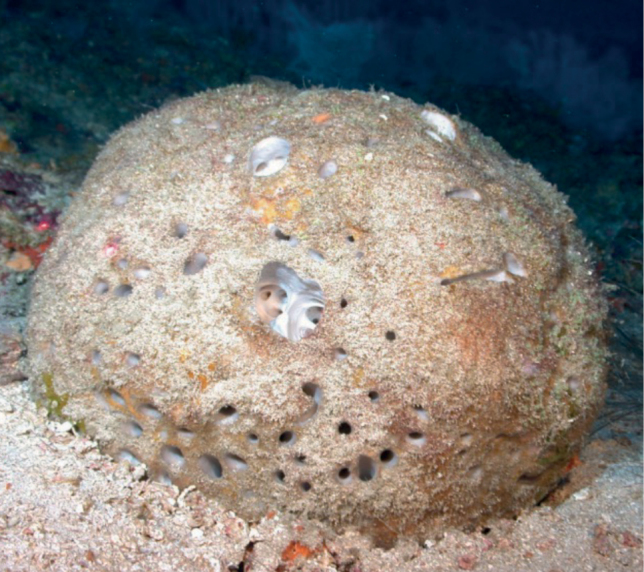
Stellettinopsiscf.megastylifera, 76 m deep. Samples DFH9-13C.

###### Distribution and abundance.

The species is reported from shallow depths growing on coral reefs, rocks, sand, or mangroves (3–25 m deep) in the Colombian Caribbean, Curacao, Panama, Belize, southern GOM, and Dominican Republic. Rare species. This is the first report of this species for the north GOM mesophotic. At FGBNMS, moderate abundance at three sites.

###### Ecology.

Coralline algae reefs, algal nodules.

###### Identification.

SWK, CA, KR.

###### References.

Sandes et al. 2020; Wintermann-Kilian and Kilian 1984.

#### ﻿Order Tetractinellida

**Sub-order Astrophorina**

**Family Geodiidae**

##### ﻿ Erylus alleni

Taxon classificationAnimaliaTetractinellidaGeodiidae

de Laubenfels, 1934

6265C994-95D9-51C7-B278-E0ECBF0187BA

Fig. 39

###### Diagnostic features.

Stalks with heart-shaped tops (3–7 cm in height, 1–3 cm wide). Dark brown in color externally, tan internally. Very smooth surface. One or two oscula per stalk located at the tips (1–5 mm wide). The oscula continued by an atrium 1–2 cm deep. Dense in consistency.

**Figure 39.**
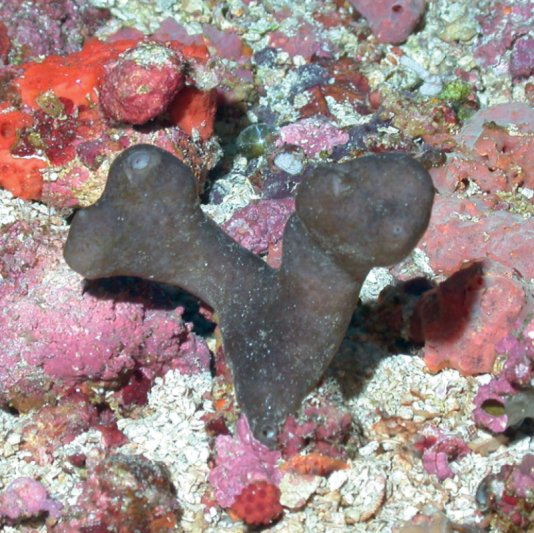
*Erylusalleni*, 60 m deep. Samples DFH9-12E, DFH9-12F.

###### Similar species.

The stalked growth form and certain spicule details (elliptical aspidasters, two categories of oxyasters) allow its differentiation with co-occurring similar species such as *Erylusgoffrilleri* and *Erylustrisphaerus*.

###### Distribution and abundance.

Mesophotic depths in Puerto Rico and Brazil. Rare species. This is the first report of this species for the GOM, at FGBNMS found with rare to low abundance (1–10) at 7 sites.

###### Ecology.

Coralline algae reefs, algal nodules.

###### Identification.

KR, CS, SWK, MCD.

###### Reference.

Mothes et al. 1999.

##### ﻿ Erylus goffrilleri

Taxon classificationAnimaliaTetractinellidaGeodiidae

Wiedenmayer, 1977

793D67A4-66D4-53C3-AC27-D3743D9968D4

Fig. 40

###### Diagnostic features.

Massive amorphous to lobate. Dark brown color that lightens towards the base of the lobes. Smooth surface, slight wrinkles when out of water. One apical oscule, on top of each lobe (4–8 mm wide), with a paler colored membrane. Compressible but dense in consistency, easily breakable.

**Figure 40.**
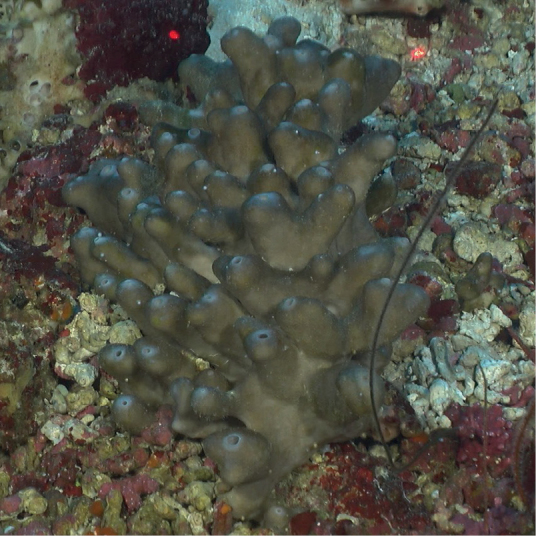
*Erylusgoffrilleri* 69 m deep. Sample GFOE3-32.

###### Similar species.

Several species of *Erylus* from the tropical western Atlantic are very similar in external appearance. The calthrop-like triaenes and the tylasters allows its distinction from co-occurring species.

###### Distribution and abundance.

Reported in shallow and mesophotic reefs in the Bahamas and Jamaica. First report of this species for the GOM at FGBNMS region, found at low abundance (1–10), at Geyer Bank.

###### Ecology.

Algal nodules.

###### Identification.

KR, CS, SWK, MCD.

###### Reference.

Wiedenmayer 1977.

##### ﻿ Erylus trisphaerus

Taxon classificationAnimaliaTetractinellidaGeodiidae

(de Laubenfels, 1953)

423E991C-3B9D-5C87-A56D-DC9CFB15F370

Fig. 41

###### Diagnostic features.

Massive amorphous to lobate, dark to paler brown. The surface is smooth with very small pores. Oscula on top of lobes, ≤ 1 cm wide, with a very thin brown membrane. Compressible and dense in consistency.

**Figure 41.**
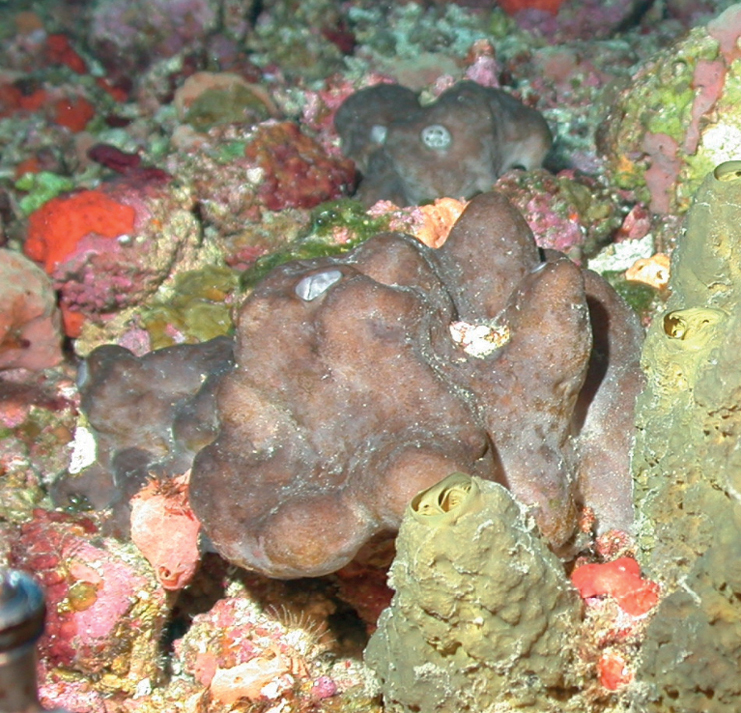
*Erylustrisphaerus*, 61 m deep. Sample DFH9-12G.

###### Similar species.

The peanut-shaped aspidasters, with two to three swollen areas, allows its distinction from co-occurring species of the genus.

###### Distribution and abundance.

This is a rare species originally described from Apalachee Bay in north Florida at 13 m deep. Since then, it has been reported from shallow reefs in Alacranes Reef (Southern GOM), Cuba, and Curacao. This is the first report from mesophotic reefs and first report from northwestern GOM at FGBNMS where it occurs with rare to low abundance (1–10) at six sites.

###### Ecology.

Coralline algal reefs, algal nodules, lower mesophotic reefs.

###### Identification.

KR, CA, SWK, MCD.

###### Reference.

Ugalde et al. 2015.

##### ﻿ Geodia cf. curacaoensis

Taxon classificationAnimaliaTetractinellidaGeodiidae

van Soest et al., 2014

52AC5E2C-4629-5975-9E10-A22E7D9E6998

Fig. 42

###### Diagnostic features.

Spherical, approx. 7 cm in diameter, with a roundish black apical plate (2 cm wide). The sponge color is pale brown with reddish tinges. The surface is mostly smooth, with patches with sediments or turf around the apical plate, and occasionally on body side. Many oscula (2 mm wide) concentrated on the apical plate. Hard as a rock. The cf. is assigned due to the black color of the sieve plate, larger oscula, and the because size of the large category of oxea of *Geodiacuracaoensis* is twice the size of oxea from the FGBNMS specimen.

**Figure 42.**
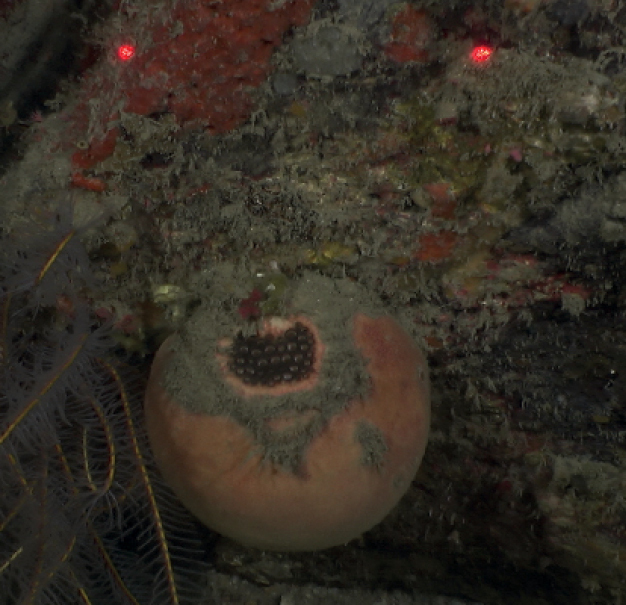
Geodiacf.curacaoensis 81 m deep. Sample GFOE3-21.

###### Similar species.

This specimen is very similar to *Geodiacuracaoensis*, in overall external morphology, and spicule composition.

###### Distribution and abundance.

*Geodiacuracaoensis* was described from mesophotic depths in Curacao and was recorded in shallow reefs at Alacranes Reef, south GOM, and at mesophotic depths in Cuba. This morphotype was found at FGBNMS in low (2–10) abundance at six sites.

###### Ecology.

Coralline algae reefs, algal nodules, lower mesophotic reefs.

###### Identification.

KR, CA, SWK, MCD.

###### Reference.

Ugalde et al. 2015.

#### ﻿Order Tetractinellida

**Sub-order Astrophorina**

**Family Theonellidae**

##### ﻿ Discodermia

Taxon classificationAnimaliaTetractinellidaTheonellidae

sp . 1

80BB54B4-4C64-5463-B60A-EE0A575B4DD0

Fig. 43

###### Diagnostic features.

Massive columnar cluster, flattened tops of columns with one apical oscule on each. Smooth to rugose surface. Tan color with pale brown tops. Columns 2–3 cm wide and 10–15 cm tall.

**Figure 43.**
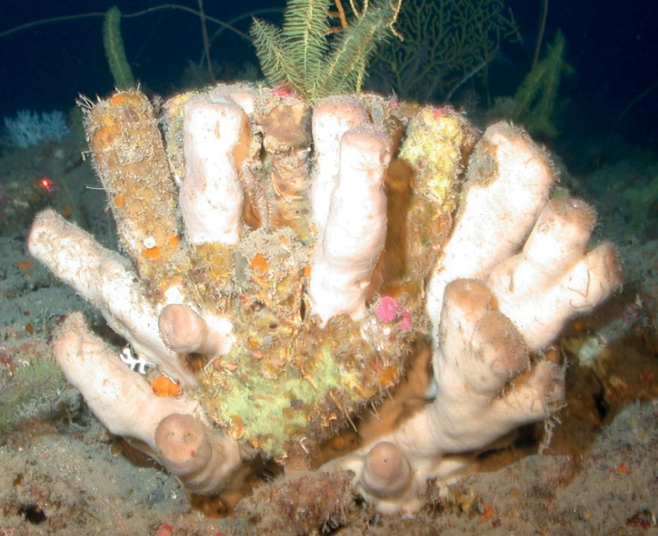
*In-situ* photograph of *Discodermia* sp. 1, 107 m deep. Photo code SP05.

###### Similar species.

The image of a *Discodermiadissoluta* specimen (van Soest et al. 2014) shows a columnar growth form for the species. Spicule preparations would be necessary to determine the species identity of this sponge.

###### Distribution and abundance.

The genus *Discodermia* is common in deep waters at the tropical western Atlantic. *Discodermiadissoluta* is the most widespread distributed species of the genus in the region from the GOM to Brazil. At FGBNMS, rare to low (1–10) abundance at two sites.

###### Ecology.

Lower mesophotic reefs.

###### Identification.

KR, CA, SWK, MCD.

###### Reference.

van Soest et al. 2014.

#### ﻿Order Tetractinellida

**Sub-order Astrophorina**

**Family Thrombidae**

##### ﻿ Yucatania sphaeroidocladus

Taxon classificationAnimaliaTetractinellidaThrombidae

(Hartman & Hubbard, 1999)

D12F4227-B529-562F-BB06-C8F5E5758D1E

Fig. 44

###### Diagnostic features.

Encrusting to massive sponge (1–9 cm in thickness). Ochre brown externally in life. The sponge has a vermetid gastropod growing within its body. Surface microhispid, rough to the touch, oscula numerous and scattered, rounded to oval, 1–4.5 mm wide. Some of the openings at the surface correspond to the vermetids.

**Figure 44.**
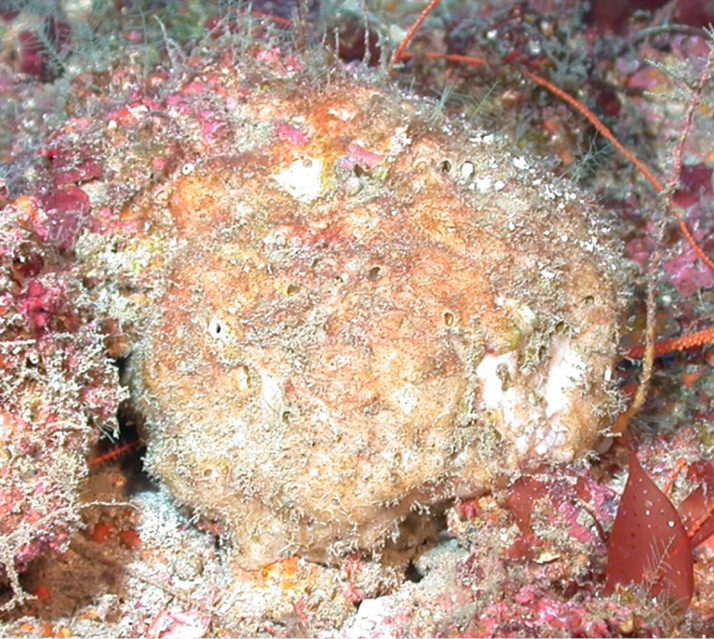
*In-situ* photograph of *Yucataniasphaeroidocladus* sp. 70–72 m deep. Samples DFH9-5C, DFH9-6D.

###### Similar species.

Any massive-amorphous to subglobular species that accumulate debris can be confused with this species. Spicule composition is essential for its identification.

###### Distribution and abundance.

Mesophotic depths from eastern Brazil, Guiana, Trinidad, and continental platform of the Yucatan Peninsula. Widely distributed at the mesophotic depths in FGBNMS (12 sites), with rare or medium abundance (1–100).

###### Ecology.

Coralline algae reefs, algal nodules, lower mesophotic reefs.

###### Identification.

KR, CA, SWK, MCD.

###### References.

Gómez 2006; Hartman and Hubbard 1999.

#### ﻿Order Tetractinellida

**Sub-order Spirophorina**

**Family Tetillidae**

##### ﻿ Cinachyrella

Taxon classificationAnimaliaTetractinellidaTetillidae

sp. 1

9F4C6ECC-9FE8-5D11-AE9C-229F673D32BF

Fig. 45

###### Diagnostic features.

Globular sponge (12 cm wide). Neon yellow in and out, covered by sediment obscuring the sponge color. The surface appears smooth, rough to the touch; microhispid microscopically. Few apical oscula (6–8 mm wide). Dense in consistency. This specimen was initially identified as *Tetilla* sp. However, an 18S barcoding study shows this species is 99.9% *Cinachyrella* sp. (Iris Segura, pers. comm., 2022). The specimen studied lacks protrienes and anatrianes found in all *Cinachyrella* species from the region. This specimen has long oxea: 2500–3000 × 10–50 µm, small oxea: 130–170 × 5 µm, and sigma (c-, and s-shaped), 20–30 µm long × < 1 µm wide. The determination of this species requires further comparative work and the analyses of other genetic markers.

**Figure 45.**
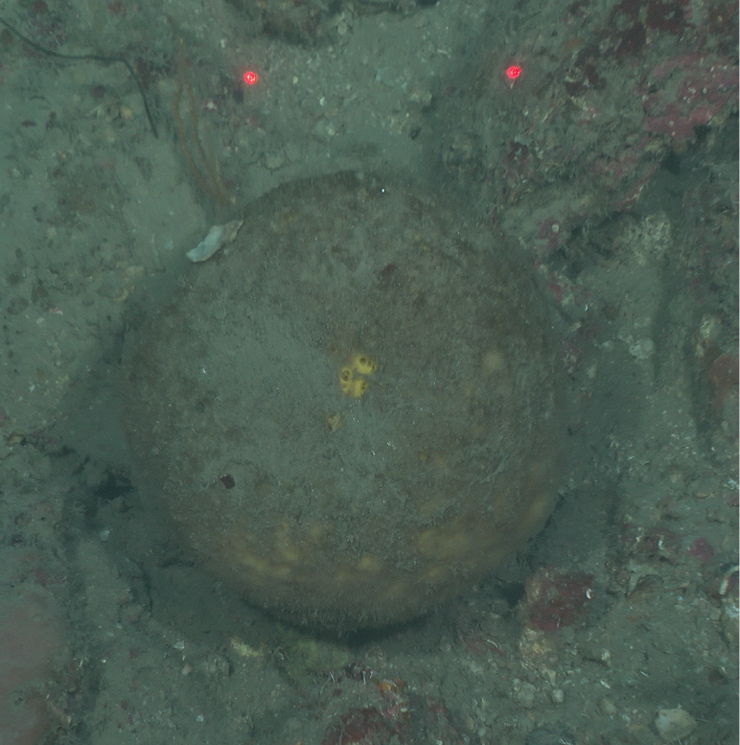
*In-situ* photograph of *Cinachyrella* sp. 1, 81 m deep. Sample GFOE3-20.

###### Similar species.

Globular yellowish species of the genera *Cinachyrella*, *Cinachyra*, or *Tetilla*.

###### Distribution and abundance.

At FGBNMS this species is rare, appearing once at one site.

###### Ecology.

Coralline algal reefs.

###### Identification.

Iris Segura, MCD.

###### Reference.

van Soest and Rützler 2002.

#### ﻿Order Chondrosida

**Family Chondrosiidae**

##### ﻿ Chondrosia collectrix

Taxon classificationAnimaliaChondrosidaChondrosiidae

(Schmidt, 1870)

EFED6122-51EB-57D4-BBB2-D6ECF4630264

Fig. 46

###### Diagnostic features.

Thick encrusting (1–3 cm in thickness) to lobate, brown, black to tan in color externally with darker spotted areas, tan internally. Smooth surface, and round oscula, with elevated membranes. This species has very cartilaginous consistency.

**Figure 46.**
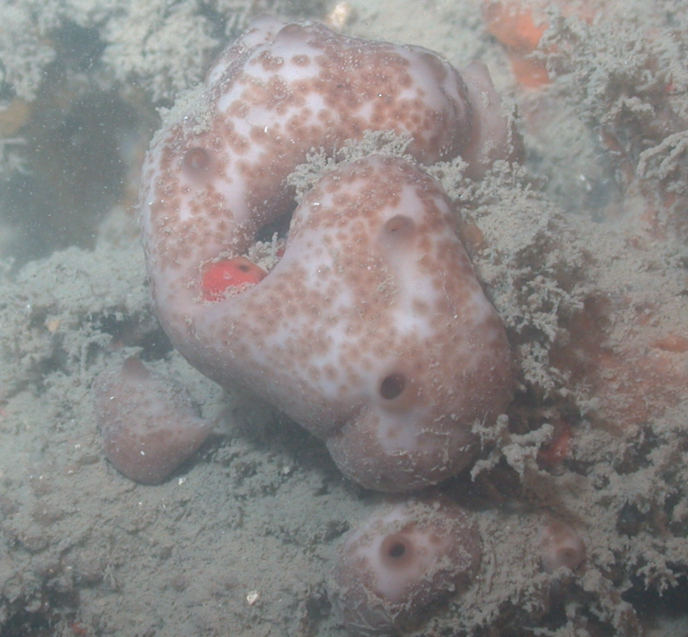
*Chondrosiacollectrix*, 60 m deep. Sample DFH9-9D.

###### Similar species.

*Chondrillacaribensis* is very similar in growth form and color but it lacks oscula with a collared membrane and possesses typical and abundant spheraster spicules.

###### Distribution and abundance.

The species is distributed at coral reefs, seagrasses, and/or mangroves in Florida, Bermuda, throughout the Caribbean, Brazil, and southern Gulf of Mexico. At FGBNMS is widely distributed with low to high abundance (10–100+) occurring at 12 sites.

###### Ecology.

Inhabits lower mesophotic reef and heavily silted reef, coralline algae reef, algal nodules.

###### Identification.

KR, CA, SWK, MCD.

###### Reference.

Wiedenmayer 1977.

#### ﻿Order Verongiida

**Family Aplysinidae**

##### ﻿ Aiolochroia crassa

Taxon classificationAnimaliaVerongiidaAplysinidae

(Hyatt, 1875)

14C7318E-FF2A-5621-81A9-D282AD4DD772

Fig. 47

###### Diagnostic features.

Massive/lobate to amorphous. Variable color from yellow to pink and purple. The surface has roundish conules. The oscula are on top of lobe(s), have small flat oscular membranes the same color than the sponge. Compressible and dense in consistency.

**Figure 47.**
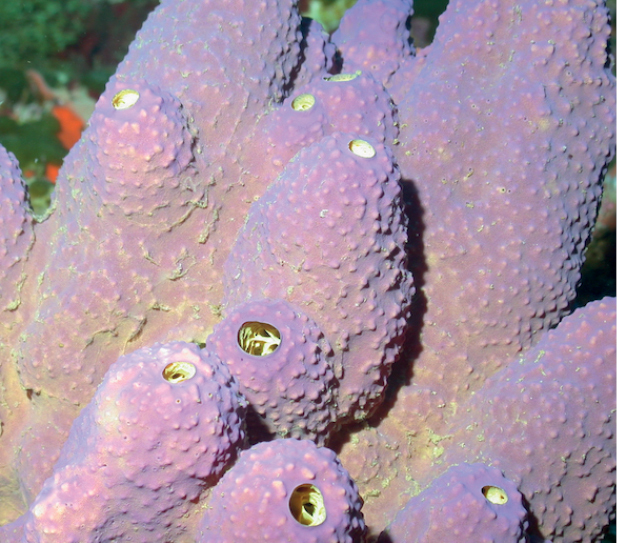
*Aiolochroiacrassa*, 52 m deep. DFH5 Dive 11269 14 17 20.JPG.

###### Similar species.

Some specimens of *Verongularigida*, with few ridges at the surface, can be confused with this species.

###### Distribution and abundance.

This species is very common at shallow coral reefs in Florida, Bermuda, Bahamas, throughout the Caribbean, Brazil, and Gulf of Mexico. At mesophotic depths in FGBNMS it is widely distributed, occurring at ten sites.

###### Ecology.

Coral communities.

###### Identification.

KR, CA, SWK, MCD.

###### Reference.

Wiedenmayer 1977.

##### ﻿ Aplysina

Taxon classificationAnimaliaVerongiidaAplysinidae

sp. 1

1A0DCD6E-50E2-5B39-A84D-9AAEACE2D46E

Fig. 48

###### Diagnostic features.

A single white tube (7 cm high, 3–4 cm wide); the sponge turns to medium brown color in alcohol. The surface is verrucose to finely conulose, and firm in consistency. One apical oscule (6 mm wide) with a thin membrane.

**Figure 48.**
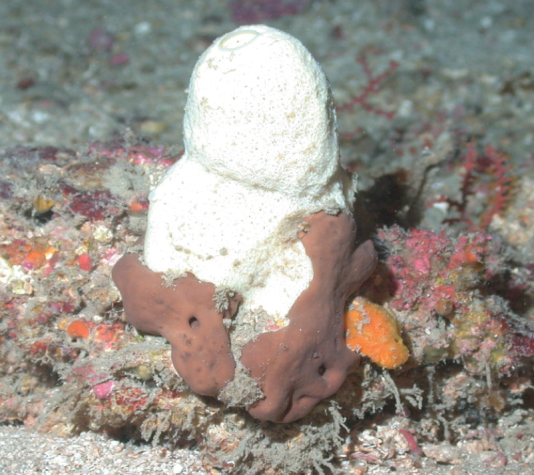
*Aplysina* sp. 1 at 88 m deep. Sample DFH9-2B.

###### Similar species.

The overall growth form of this species is similar to that of *Aplysinaarcheri*. However, *Aplysinaarcheri* tubes are usually much larger, and invariably a pink to violet color at all its depth range of distribution. A similar whitish *Aplysina* with larger dimensions was observed in Cuba at 58 and 81 m deep.

###### Distribution and abundance.

This is a unique and rare species of *Aplysina*, found in mesophotic reefs at FGBNMS and Pulley Ridge. At FGBNMS it was found once at the east Flower Gardens bank.

###### Ecology.

Found at coralline algal reef, algal nodule, lower mesophotic reef.

###### Identification.

KR, CA, SWK, MCD.

###### References.

van Soest 1978; Pinheiro et al. 2007.

##### ﻿ Aplysina cf. archeri

Taxon classificationAnimaliaVerongiidaAplysinidae

(Higgin,1875)

F6E28D0E-EE4D-5616-867E-570C6AC00F20

Fig. 49A, B

###### Diagnostic features.

Clusters of short tubes that spread laterally. SP04 was 10–20 cm high, and 3–5 cm in diameter, and SP25 was 3–10 cm high 3–6 cm wide, pink to deep purple in color. Fistulose rods sporadically grow out from the tubes. Surface rugose and microconulose. Roundish tube tops, more pronounced on SP25. The specimens are tentatively identified from the photographs as Aplysinacf.archeri due to the predominance of short roundish tubes, and lateral growth; no samples were available for analysis.

**Figure 49.**
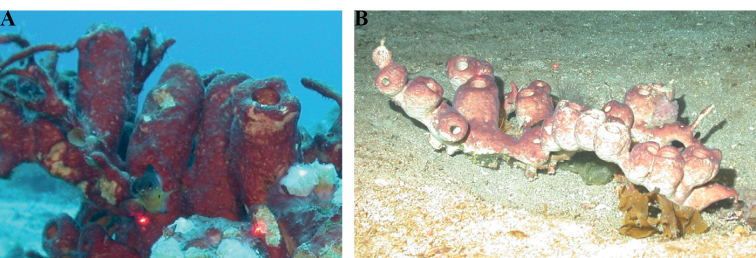
Aplysinacf.archeri, at 43–76 m deep. Photo code **A** SP04 and **B** SP25.

###### Similar species.

At least three species of *Aplysina* can be a cluster of short tubes, viz. *A.fistularis*, *A.insularis*, and *A.muricyana* but their color is mostly yellow. Collection and genetic data would be very helpful to discern these species.

###### Distribution and abundance.

*Aplysinaarcheri* is common at shallow coral reefs in Florida (Dry Tortugas) and throughout the Caribbean. This species might be a morphological variant of the species or a closely related species which occurs as a single or clumps of elongated purplish tubes.

###### Ecology.

Coral communities, sandy substrates.

###### Identification.

MCD.

###### References.

van Soest 1978; Pinnheiro et al. 2007.

##### ﻿ Verongula rigida

Taxon classificationAnimaliaVerongiidaAplysinidae

(Esper, 1794)

53279E26-061C-5E24-9B6B-E42C0F12FE57

Fig. 50

###### Diagnostic features.

Massive lobate to tubular species. This sample had multiple tubes of different lengths (4–20 cm high, 2–4 cm wide). Reddish yellow in color when alive, purple as dry or in alcohol. The surface is rugose to verrucose, ribbed, but smooth to the touch, not like sandpaper. One oscule (0.8–1 cm) on top of each tube, the opening extending the length of sponge. Oscula with a flat diaphragm-like contractile membrane darker in color. The consistency is firm but compressible, fibrous, and tough.

**Figure 50.**
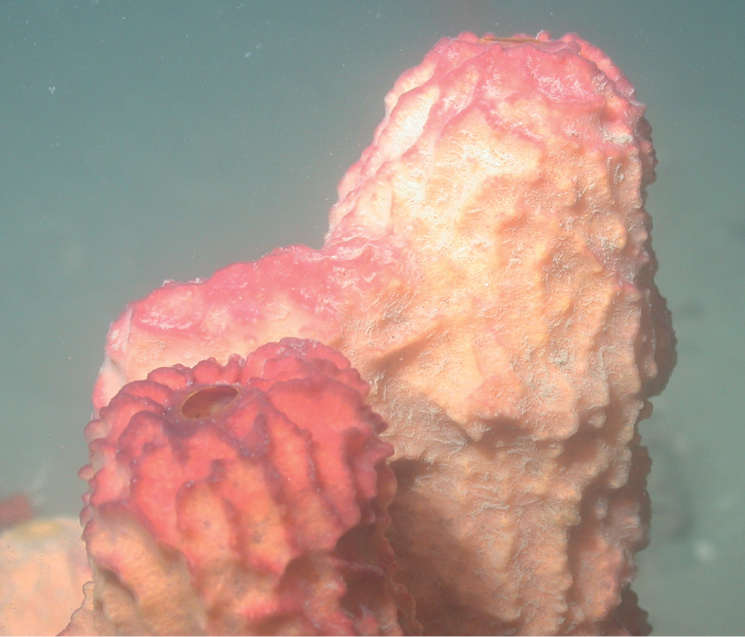
*Verongularigida* at 60 m deep. Samples DFH8-38B, DFH9-9B.

###### Similar species.

Specimens of this species with slightly ribbed surface can be confused with *Aiolochroiacrassa*. Some morphotypes of *Smenospongiaaurea* can also look like short tubes of *Verongularigida*.

###### Distribution and abundance.

This species is common at shallow coral reefs throughout the Caribbean. At FGBNMS, rare, found only at three sites.

###### Ecology.

Heavily silted lower mesophotic reefs.

###### Identification.

KR.

###### Reference.

Wiedenmayer 1977.

##### ﻿ Verongula reiswigi

Taxon classificationAnimaliaVerongiidaAplysinidae

Alcolado, 1984

3477D070-F210-563F-A140-8C4402D06330

Fig. 51

###### Diagnostic features.

Large tube or vase, wider at the base or at the mid body. The color is yellow with green or pinkish tinges alive, purple when dry or in alcohol. The outer surface is ribbed, forming a regular pattern that covers the whole sponge surface. One large opening (oscule or pseudo-oscule) at top of each “vase” (> 5 cm), with a very thin membrane (1–2 mm) that surrounds the whole rim. The consistency is firm but compressible.

**Figure 51.**
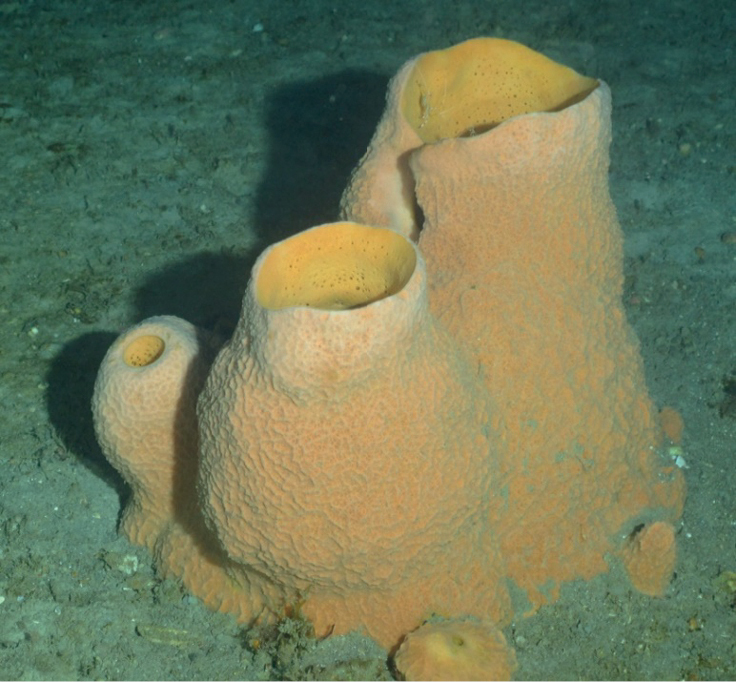
*Verongulareiswigi* at 76 m deep. Photo code SP58.

###### Similar species.

The overall shape, “oscule” size, and surface of this species makes it unique.

###### Distribution and abundance.

This species is rare in occurrence at shallow and mesophotic coral reefs in Florida, Bahamas, Cuba, and eastern Caribbean. Rare at FGBNMS, seen only once at one site,

###### Ecology.

Heavily silted lower mesophotic reef.

###### Identification.

MCD.

###### References.

Alcolado 1984; Perez et al. 2017.

#### ﻿Order Verongiida

**Family Ianthellidae**

##### ﻿ Ianthellide

Taxon classificationAnimaliaVerongiidaIanthellidae

sp. 1.

B30E12BF-FC77-5F20-8AFC-B985CB660C81

Fig. 52

###### Diagnostic features.

**Figure 52.**
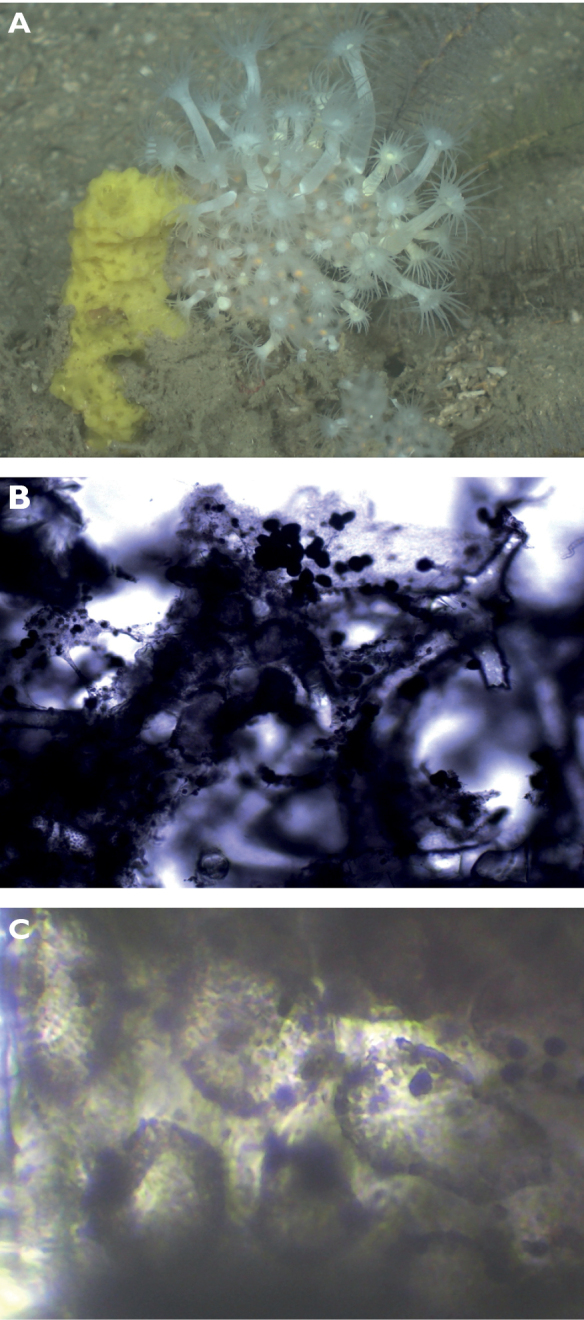
**A***In-situ* photograph of a thin yellow Verongid growing on an hexactinellid skeletal framework highlighted by the red arrow, 147 m deep. Sample GFOE3-23A **B** fragment of sponge observed under light microscope 100X; arrows show large dark cells potential spherulous cells (SC), and smaller dark cells, potentially granular cells (GC) **C** smear of a sample fragment observed with a light microscope at 400× magnification. Note the large ovoid choanocyte chambers (red arrow).

Thin encrusting sponge, < 1 mm thick, bright yellow in life, purple in alcohol (Fig. 52A, red arrow). This thin sponge lacked any type of skeleton and seems to be overgrowing the skeleton of a dictyonal hexactinellid skeletal framework (Fig. 52B). No microscleres could be seen when dissecting and analyzing the skeleton of the hexactinellid, thus it could be dead. The conspecificity of the hexactinellid with Iphiteonpanicea sample GFOE3-23, to the right of this yellow sample (GFOE3-23A) could not be identified. The thin sponge appears undetachable from the skeletal framework where it grows. Dark cells similar to verongiid spherulous cells (SC), and granular cells (GC), and wide elliptical choanocyte chambers (CC), 30–80 µm in diameter, support the interpretation of this species as a fiber-less species of the family Ianthellidae (Fig. 52B, C). This is the first report of a verongiid overgrowing an hexactinellid.

###### Similar species.

Two Ianthellidae genera without fibers have been described, *Hexadella* and *Vansoestia*, which have species with thin bodies, and yellowish color. However, those species have a more detachable leathery body, with surfaces ornamented by dermal canals, and prominent oscula.

###### Distribution and abundance.

At FGBNMS the species was collected once at Elvers Bank.

###### Ecology.

Coralline algae reefs, lower mesophotic reefs, algal nodules.

###### Identification.

MCD.

###### Reference.

Díaz et al. 2015.

#### ﻿Order Dendroceratida

**Family Darwinellidae**

##### ﻿ Aplysilla aff. sulfurea

Taxon classificationAnimaliaDendroceratidaDarwinellidae

Schulze, 1878

221C40AC-38B1-544B-A2DE-C0EFAF437E5A

Fig. 53

###### Diagnostic features.

Thin encrusting sponge, 1–2 mm when preserved in alcohol. Pale yellow/orange in color alive. In alcohol it turns dark purple. In life the surface bears low conules, and oscula a few mm wide.

###### Similar species.

The overall growth form, color, and color change in alcohol is similar to those in *Aplysillasulfurea*.

###### Distribution and abundance.

*Aplysillasulfurea* is the type species for the genus and was described from the Adriatic and Mediterranean seas and eastern Atlantic. The reports from Bermuda, Florida, and southern Africa probably represent different species of similar habit and color. At FGBNMS the species is widely distributed at 14 sites with a range of abundance from rare to medium (2–100).

**Figure 53.**
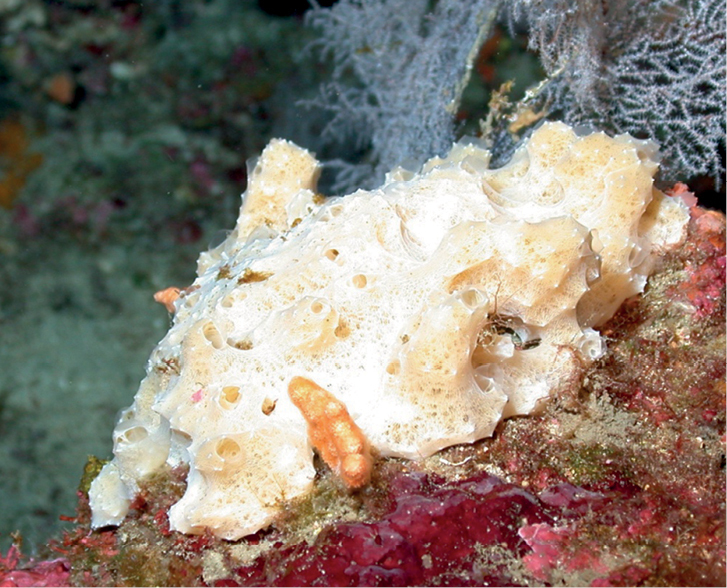
*Aplysilla*aff. *sulfurea*, 78 m deep. Samples DFH9-14B.

###### Ecology.

Coralline algae reefs, lower mesophotic reefs, algal nodules.

###### Identification.

CA, MCD.

###### References.

de Laubenfels 1950, 1953.

#### ﻿Order Dictyoceratida

**Family Dysideidae**

##### ﻿ Dysidea

Taxon classificationAnimaliaDictyoceratidaDysideidae

sp. 1

8B97DB54-C8B2-56B4-8686-BD6366536F7B

Fig. 54

###### Diagnostic features.

Encrusting to massive (2–4.5 cm in thickness). Pale yellow to orange color in life. The surface is porous and with low conules. Many oscula with transparent membranes. The sponge is compressible.

**Figure 54.**
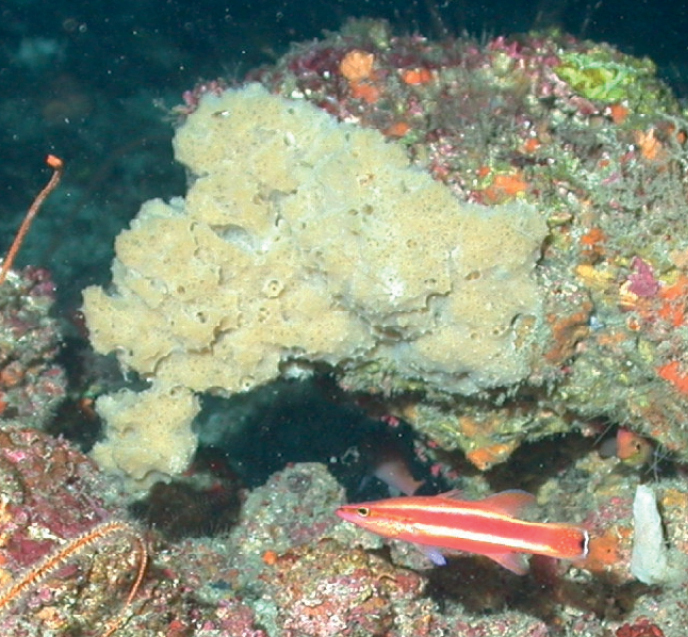
*Dysidea* sp. 1, 76 m deep. Sample DFH9-14F.

###### Similar species.

This species is similar to the Mediterranean species *Dysideafragilis*. There is an inaccurate citation of *Dysideafragilis* by de Laubenfels (1953) from the GOM, and this is probably an undescribed species.

###### Distribution and abundance.

At FGBNMS the species is widely distributed at ten sites with a range of abundance from rare (1 per site) to common (11–100).

###### Ecology.

Coralline algae reef, lower mesophotic reef, and algal nodules.

###### Identification.

KR, MCD.

###### Reference.

de Laubenfels 1953.

##### ﻿ Pleraplysilla

Taxon classificationAnimaliaDictyoceratidaDysideidae

sp. nov. 1

A472CE85-7760-543D-B978-07368A6B30F2

Fig. 55

###### Diagnostic features.

Very thin crust, pale pink color. The surface is smooth with irregularly distributed small conules (< 1 mm high). Oscula with a collar membrane and thick canals that run towards the oscula. The conules are produced by single or branching fibers that depart from a spongin basal plate.

**Figure 55.**
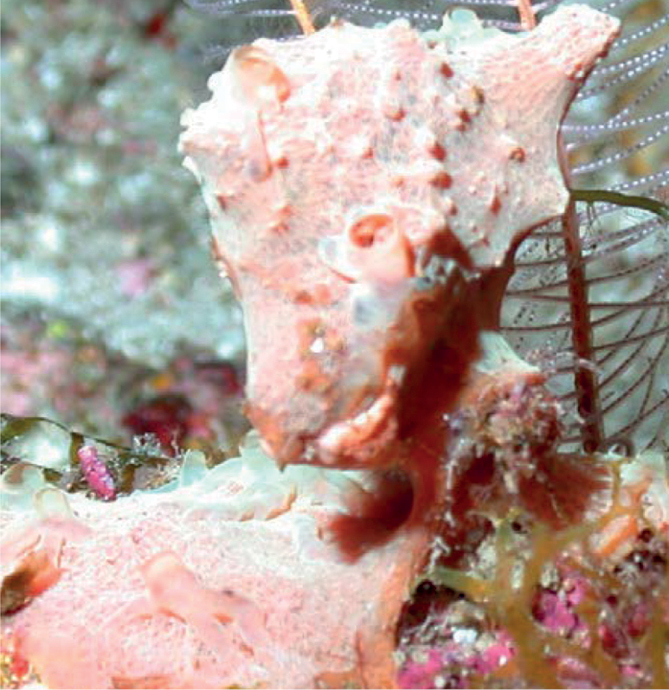
*In-situ* photo of *Pleraplysilla* sp. 1, 79 m deep. Sample DFH9-14C.

###### Similar species.

Very similar in external appearance to *Vansoestiacaribensis*, a skeleton-less sponge of the family Ianthellidae, Order Verongiida. This species has abundant single or dendritic fibers, dark in color with an apparent pith, and some foreign spicules inside. This sponge is very similar to the species of *Pleraplysilla* depicted by Zea et al. (2014). It is currently an undescribed species.

###### Distribution and abundance.

Reported by Zea et al. (2014) from the Bahamas and possibly Boynton Beach, FL. At FGBNMS the species is found at seven sites with an abundance ranging from rare to low (1–10) at six sites, to common (11–100) at one site.

###### Ecology.

Coralline algae reefs, lower mesophotic reefs.

###### Identification.

KR, SK, CA, MCD.

###### Reference.

Zea et al. 2014.

##### ﻿ Pleraplysilla

Taxon classificationAnimaliaDictyoceratidaDysideidae

sp. nov. 2

BCA2B2F5-A475-5104-B2CD-E93B0C6F4DF1

Fig. 56

###### Diagnostic features.

Massive bushy, 5 cm wide, 7 cm high. Tan in color alive. Sharp conules, 2–3 mm high, separated by 3–5 mm. The sponge is compressible but firm. The sponge has dendritic fibers, pale in color, which incorporate broken spicules. This sponge is currently identified as an undescribed species of *Pleraplysilla*. 28S analysis of this specimen clearly places it within the Order Dictyoceratida, but not within the Family Dysideidae. 18S analysis places it as 99.5% similar to *Pleraplysillaspinifera*, the type of the genus; however, that is an eastern Atlantic and Mediterranean species. This result supports the generic assignation of this new species from GOM (Diaz, Segura, and Pomponi, unpublished data).

**Figure 56.**
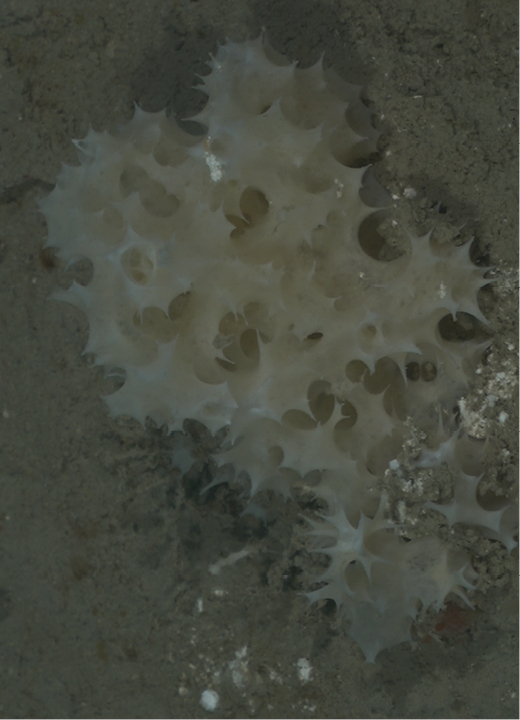
*Pleraplysilla* sp. 2, 47 m deep. Sample GFOE3-19.

###### Similar species.

Its massive habit is similar to that of the shallow Caribbean mangrove species *Pleraplysillastocki*, and to *Pleraplysillaspinifera* from the Mediterranean.

###### Distribution and abundance.

The species has been collected once from FGBNMS, and once from Pulley Ridge, at the southeast GOM (MCD).

###### Ecology.

Coralline algal reef.

###### Identification.

Iris Segura, MCD.

###### Reference.

van Soest 1978.

#### ﻿Order Dictyoceratida

**Family Irciniidae**

##### ﻿ Ircinia campana

Taxon classificationAnimaliaDictyoceratidaIrciniidae

(Lamarck, 1814)

3F6B6018-3EC9-5AD0-9D78-A9D7E1FECFE8

Fig. 57

###### Diagnostic features.

Flabellate to fan- or cup-shaped, sometimes pedunculated. Brown, gray, pinkish, or white color in life. The surface is regularly conulose and rugose. Abundant round oscula (4–8 mm) on the inner wall surface.

**Figure 57.**
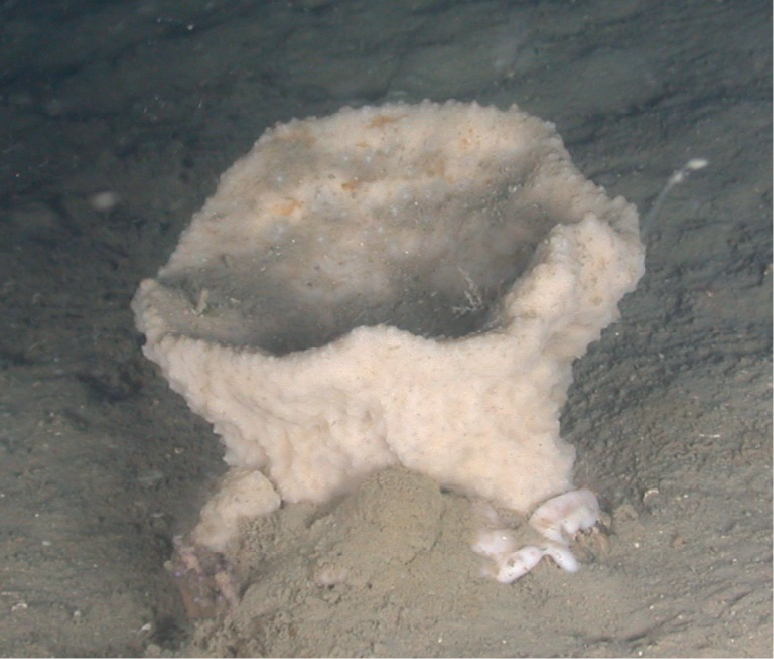
*Irciniacampana*, 56 m deep. Photo code SP10.

###### Similar species.

This might be a different species from the shallow reef species, but close genetic and morphological comparison must be carried out to distinguish *Ircinia* species (Kelly and Thacker 2021).

###### Distribution and abundance.

Widespread throughout the Caribbean at shallow coral reefs and seagrass meadows. This is the first report of the species for the northwestern GOM and at mesophotic depths in the GOM. At FGBNMS, low (1–10) in abundance and only documented at Stetson Bank. Díaz et al. 2021 report this species' morphotype at mesophotic depths (50–79 m) in various MPA's from North Carolina, South Carolina and Florida.

###### Ecology.

Lower mesophotic reefs, coralline algae reefs.

###### Identification.

MCD.

###### Reference.

Díaz et al. 2019.

##### ﻿ Ircinia cf. campana

Taxon classificationAnimaliaDictyoceratidaIrciniidae

(Lamarck, 1814)

3CBC4EB4-9069-5081-97FD-FF99ED6CF6A6

Fig. 58

###### Diagnostic features.

Flabellate to fan. Brown, gray, to pinkish in color. The surface is regularly conulose. Abundant round oscula (2–3 mm) on the upper surface, sometimes clumped. The cf. is to highlight the uncommon plate-shape habitus for the species, indicating that this morphotype could represent either a different species or a variant of *Irciniacampana*. Further genetic and morphologic comparisons are required.

**Figure 58.**
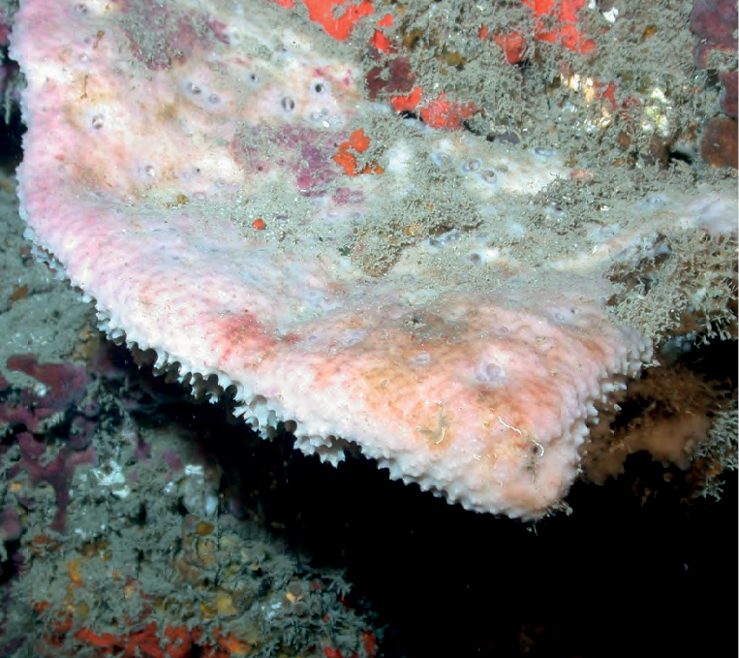
Irciniacf.campana, 55 m deep. Sample DFH9-8A.

###### Distribution and abundance.

Widespread throughout the Caribbean on shallow coral reefs and seagrass meadows. This is the first report of the species for the northwestern GOM and at mesophotic depths. Single specimen found at one locality.

###### Ecology.

Coralline algal reefs.

###### Identification.

MCD.

###### References.

van Soest 1978; Díaz et al. 2019.

##### ﻿ Ircinia strobilina

Taxon classificationAnimaliaDictyoceratidaIrciniidae

(Lamarck,1816)

8C86BB5F-D5CA-5870-A31E-F6A7A048CEBD

Fig. 59

###### Diagnostic features.

The sponge is sub-globular to massive and cake-shaped, gray to black color in life. Large specimens show an upper depression where oscula abound. The surface has characteristic large conules (2–15 mm high, 5–15 mm apart). Oscula 4–10 mm in diameter, either single or in groups, with a thin membrane. The specimens are tough in consistency.

**Figure 59.**
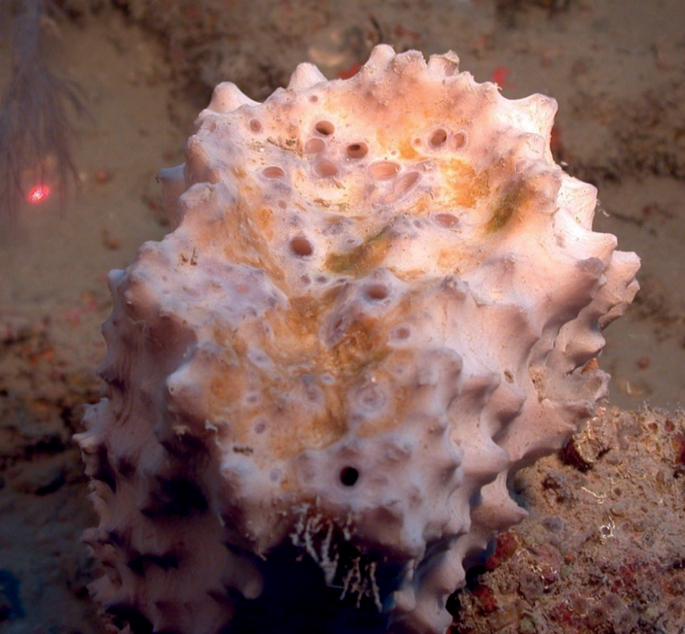
*Irciniastrobilina*, 50 m deep. Photocode SP09.

###### Distribution and abundance.

Widespread throughout the Caribbean, Bermuda, GOM, and Brazil. The species has been previously reported in the northern and southern GOM (de Laubenfels 1936; Green et al. 1986; Gómez 2007). This species is a common inhabitant of coral reefs in the southern GOM (Ugalde et al. 2021). At FGBNMS the species is abundant at Stetson and Sonnier banks.

###### Ecology.

Coral communities, coralline algae reefs, lower mesophotic reefs.

###### Id.

MCD.

###### Reference.

van Soest 1978.

##### ﻿ Ircinia

Taxon classificationAnimaliaDictyoceratidaIrciniidae

sp. 1

0B68CC98-1F28-523F-8571-6CF9616DE5A7

Fig. 60

###### Diagnostic features.

**Figure 60.**
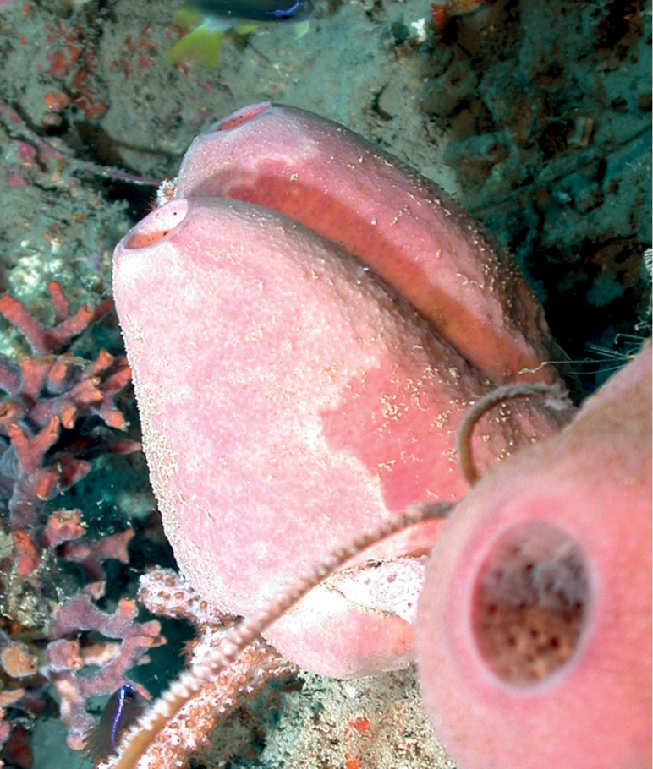
*Ircinia* sp. 1, 54–60 m deep. Samples DFH9-8B, DFH9-7F.

Single, two, or three tubes connected at the base. Tubes taper towards the tip. Pink to white in life. The tubes in Fig. 60 are 13 cm high. The surface has minute conules homogeneously spaced. One large oscule per tube (~ 2 cm in diameter) with a thin paler membrane.

###### Similar species.

This is a very unique *Ircinia* species, probably undescribed.

###### Distribution and abundance.

This is an undescribed species of *Ircinia*. At FGBNMS the species was seen once at two localities. MCD has seen this species once in the Bahamas.

###### Ecology.

Silted coralline algae reefs, silted lower mesophotic reefs.

###### Identification.

CA, KR, SK, MCD.

###### Reference.

van Soest 1978.

##### ﻿ Ircinia

Taxon classificationAnimaliaDictyoceratidaIrciniidae

sp. 2

983AA1F4-AD2F-577D-8341-8F601F6E963A

Fig. 61

###### Diagnostic features.

Cushion shape to massive (5 cm thick). The surface is finely conulose (< 1 mm high, and 1–2 mm apart). Color alive is pink to reddish brown externally, tan internally. Small oscula 2–4 mm in diameter) with a thin white membrane around their rim, sparsely distributed on the sponge. The sponge is compressible but tough to cut.

**Figure 61.**
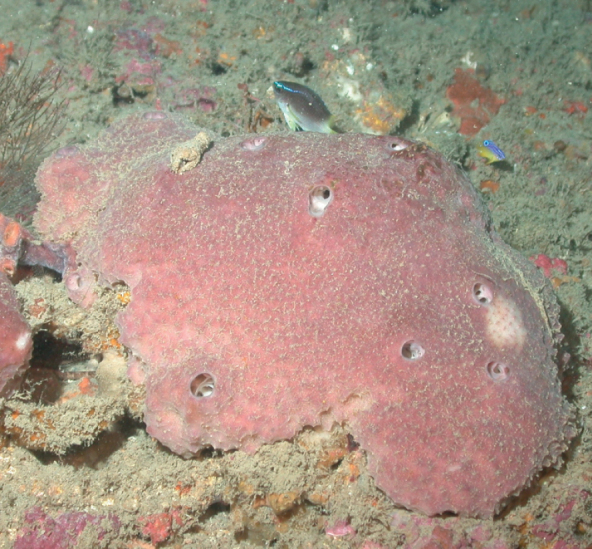
*Ircinia* sp. 2, 55 m deep. Sample DFH9-9A.

###### Similar species.

The species appears similar to *Neopetrosiaproxima* and its closest species. *Neopetrosiaproxima* lacks the conules, has a harder consistency, and a spicular skeleton, while *Irciniapossess* a skeleton of spongin fibers and filaments that may incorporate foreign particles (sand, and broken spicules).

###### Distribution and abundance.

At FGBNMS the species was found once at one site.

###### Ecology.

Coralline algal reefs.

###### Identification.

CA, KR, SK, MCD.

###### Reference.

van Soest 1978.

#### ﻿Order Dictyoceratida

**Family Thorectidae**

##### ﻿ Smenospongia cf. echina

Taxon classificationAnimaliaDictyoceratidaThorectidae

(de Laubenfels, 1934)

1DD2F15E-9E2C-58BD-8632-4D4445BB07C4

Fig. 62

###### Diagnostic features.

Globular to cushion shape, dirty yellow to grayish in life, brownish purple in alcohol. The surface has shallow roundish warts (≤1 cm wide) but feels smooth to the touch. Oscula from 2 mm to > 1 cm wide, with a slightly elevated membrane.

**Figure 62.**
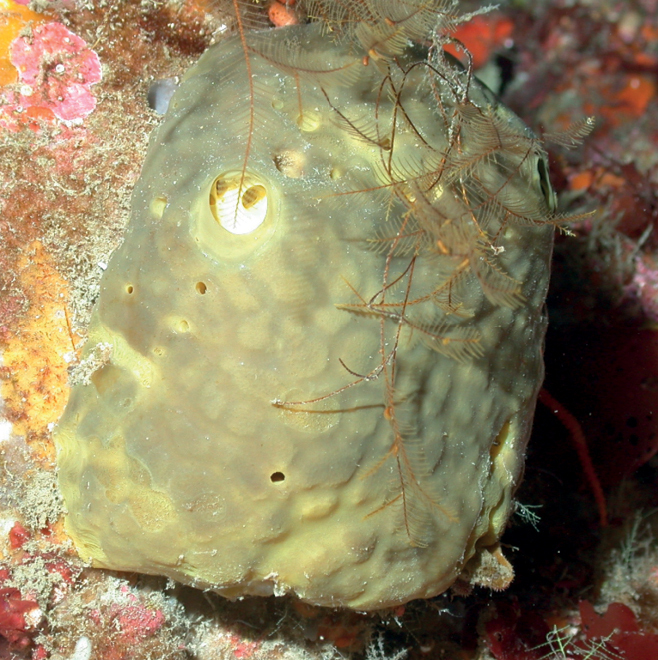
Smenospongiacf.echina, 60-69 m deep. Samples DFH9-10E, DFH9-6C.

###### Similar species.

Similar to verongiid species, *Smenospongia*spp. tends to turn purplish after collection.

###### Distribution and abundance.

*Smenospongiaechina* occurs in low abundance at shallow and mesophotic reefs in Puerto Rico (60–72 m), Belize, Florida (Dry Tortugas), Cayman Islands, and Cuba. At FGBNMS the species occurs in rare to low abundance (1–10) at four sites.

###### Ecology.

Lower mesophotic reefs, coralline algae reefs.

###### Ideentification.

CA, KR, SK, MCD.

###### Reference.

van Soest 1978; Rützler et al. 2014.

#### ﻿Class Homoscleromorpha

**Order Homosclerophorida**

**Family Plakinidae**

##### ﻿ Plakortis cf. zyggompha

Taxon classificationAnimaliaHomosclerophoridaPlakinidae

(de Laubenfels, 1934)

F81581C9-78E4-58ED-9504-52BDD4892605

Fig. 63

###### Diagnostic features.

Thick encrusting (5–10 mm thick). Pinkish brown in life. The surface is very smooth, velvety to the touch. Dense consistency. Sponge was overgrowing the base of an albino *Aplysina*spp. (DFH9-2B). Spicules larger than those of *Plakortiszyggompha* (de Laubenfels, 1934).

**Figure 63.**
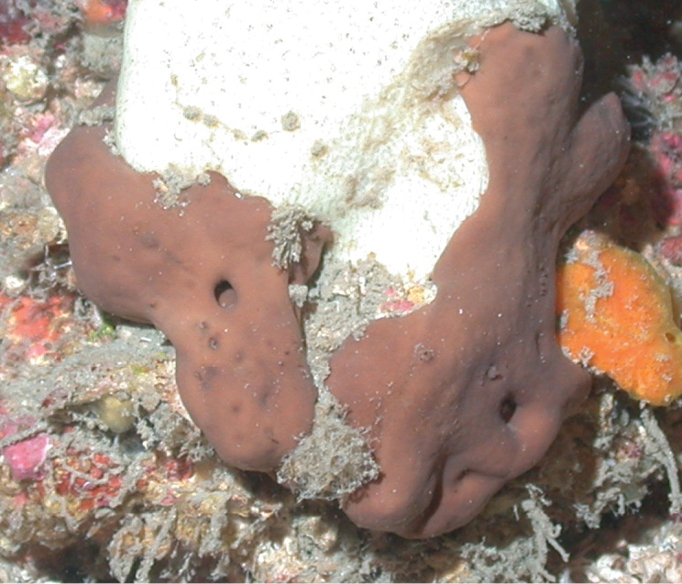
Plakortiscf.zyggompha, 88 m deep. Sample DFH9-2B.

###### Similar species.

*Plakortisangulospiculatus* and *Plakortishalichondroides*, with the same dark brown color and thick crustose shape; *Plakortiszyggompha* is always much thinner (< 5 mm) and oscula are much smaller. A genetic comparison would clarify the taxonomic status of the FGBNMS material.

###### Distribution and abundance.

The species is originally described from mesophotic depths (84–165 m), and it is also reported from Florida (Dry Tortugas), Belize (cryptic habitats), and Jamaica (mangroves). At FGBNMS the species was rare and found at only three sites.

###### Ecology.

Algal reef, algal nodule, lower mesophotic reef.

###### Identification.

SK, KR, CA.

###### References.

de Laubenfels 1934; Rützler et al. 2014.

#### ﻿Class Hexactinellida

**Order Hexasterosphora**

**Family Dactylocalycidae**

##### ﻿ Dactylocalyx pumiceus

Taxon classificationAnimaliaHexasterosphoraDactylocalycidae

Stutchbury, 1841

95C8DF50-218D-51B8-965F-82DE06BD218B

Fig. 64

###### Diagnostic features.

Basal funnel expanded distally forming a plate, or a cup, with a wavy rim. Inner cup surface has elongated pits or grooves, several cm long, < 1 cm wide. This sample has not yet been examined but the overall growth form points to this species.

**Figure 64.**
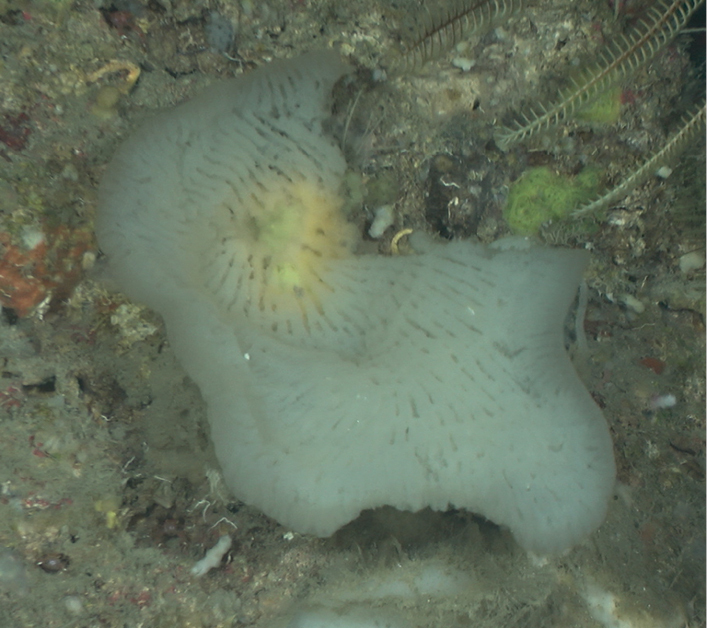
*In-situ* photo of *Dactylocalyxpumiceus*, 147 m deep. Sample GFOE3-24.

###### Distribution and abundance.

This is a species with great latitudinal distribution from Florida and Gulf of Mexico to Brazil, distributed along the western coast of the Atlantic Ocean between 300N and S, at depth of 91–1996 m. The species is also reported off the coast of Portugal. At FGBNMS the species was collected at Elvers Bank where it was found in low (1–10) abundance.

###### Ecology.

Lower mesophotic reef.

###### Identification.

MCD.

###### Reference.

Reiswig 2002.

##### ﻿ Iphiteon panicea

Taxon classificationAnimaliaHexasterosphoraDactylocalycidae

Bowerbank, 1869

D963189C-E422-572C-BE5E-45EFFBF61AEB

Fig. 65

###### Diagnostic features.

**Figure 65.**
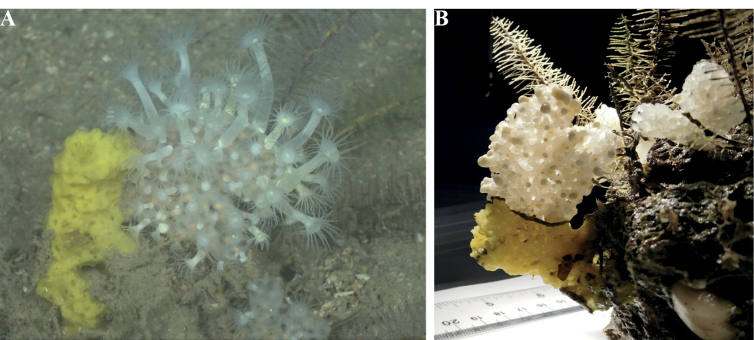
**A***In-situ* photo of 147 m deep. Samples GFOE3-23 (white) and GFOE- 23A (yellow) **B** lab photo of the specimen.

Massive, flabellate, white, glass sponge, attached to a rock. The white elongated zoanthid, *Vitrumanthusschrieri*Kise et al., 2022, was partially overgrowing its surface. The skeleton study of the white hexactinellid (GFOE- 23) revealed a dictyonal, siliceous, rectangular to triangular framework, and spicules that agree with Iphiteonpanicea as described by Reiswig (2002: 1299). What appears to be a portion of this specimen with a bright yellow color in life turned dark purple in alcohol, and it was stored as a different sample (GFOE3- 23A). Under a light microscope, the bright yellow hexactenillid appears to be a Dactylocalycidae skeletal framework, covered by thin tissue with no fibers or spicules (Fig. 51B, C). The color pattern in life and in alcohol and the type of cells and chambers suggest that this yellow tissue might represent a skeleton-less verongiid of the family Ianthellidae. The hexactinellid portion of yellow color area lacked any microscleres; this would suggest that the hexactinellid might have been dead, which would make the yellow species a potential epibiont for this hexactinellid. More study is required to clarify the identity of this apparent yellow hexatinellid. The trabecular surface is evident on the deck photograph (Fig. 65B) with round to elongate pits or grooves (2–10 mm in diameter), possibly exhalant apertures (Reiswig 2002).

###### Similar species.

When zoanthids are extended the species can look like the hexactinellid *Verrucocoeloidealiberatorii* Reiswig & Dohrmann, 2014.

###### Distribution and abundance.

This species has a northwestern Caribbean distribution (88–1957 m deep). At FGBNMS the species was collected once at Elvers Bank.

###### Ecology.

Lower mesophotic reefs, sandy bottoms. The zoanthid *Vitrumanthusschrieri* (Parazoanthidae) was originally described in association with the glass sponge *Verrucocoeloidealiberatorii*. In this sample, the identity of the zoanthid was obtained by barcoding data (28S gene) (Iris Segura, pers. comm., 2022).

###### Identification.

MCD.

###### References.

Kise et al. 2022; Reiswig and Dorhrmann 2014; Reiswig 2002.

## ﻿Discussion and conclusions

This checklist of 64 sponge species represents only a portion of the sponge fauna inhabiting mesophotic depths in the Flower Garden Banks National Marine Sanctuary region. Caribbean coral reefs that have been studied for years, including surveys of both open and sciophilous (shaded) habitats, such as the Belizean barrier reef (Rützler et al. 2014) or the Netherland Antilles (Meesters et al. 1991; van Soest 1978, 1980, 1984; van Soest et al. 2014), describe species richness at more than 200 sponge species. Considering the high diversity of habitats and substrates in the studied region, it is expected that a similar sponge biodiversity potential is possible at the Flower Garden Banks National Marine Sanctuary. Therefore, the 64 species in this study likely represent no more than one-third of the potential sponge biodiversity in the region, and are focused on some of the most conspicuous components in the sanctuary. Even with this partial representation, there are 15 species that could only be identified to genus level, and one just to family level, demonstrating the high potential to find new species at these mesophotic depths in the northwestern Gulf of Mexico.

Our most recent collection conducted off the Sanctuary boundaries in 2019 contributed specimens of seven potentially new species of the genera *Auletta*, *Petrosia*, *Xestospongia*, *Cinachyrella*, *Siphonodictyon*, and *Pleraplysilla*, and one specimen from the family Ianthellidae. These include species with significant biomass representation and widespread occurrence such as *Petrosia*sp. nov. 1 and *Xestospongia*sp. nov. 1, or species with novel or important ecological features such as the skeleton-less Ianthellidae sp. 1 overgrowing an Hexactinellid, and the bioeroding species *Siphonodictyon*sp. nov. 1. Molecular analyses (using 28S and 18S genes) are in progress to complement the morphological characteristics to refine and, in some cases, confirm the identification of these potentially new mesophotic sponge species. Part of the data from this concurrent study was essential for the generic determination of three of the species included in this paper, *Cinachyrella* sp. 1, *Pleraplysilla*sp. nov. 2, and *Siphonodictyon*sp. nov. 1.

The biological role of sponges in coral ecosystems should ignite interest to continue studying this fauna from less studied mesophotic habitats. Sponges are known for their high diversity and high biomass in shallow and mesophotic coral reefs (Díaz and Rützler 2001; Reed et al. 2018). They are important space competitors either by occupying the substrate or by overgrowing other reef organisms (Aerts 1998; Pawlik 2011), and they provide habitats for hundreds of species within or around them (Villamizar and Laughlin 1991). Spongivory is a well-known relationship with a variety of reef fauna ranging from turtles and fish to sea stars (Wulff 1994; Bell 2008; Mah 2020) and sea slugs (Mullins 2021). Sponges, through their high capacity of water filtration and their associated microbes, mediate several microbial metabolic processes such as photosynthesis, nitrification, nitrogen fixation, denitrification, sulfate reduction, and anaerobic ammonium oxidation (anammox) (Fiore et al. 2013). Several species (e.g., *Ircinia*) are known to accumulate phosphorus in granules. Therefore, sponges are known to be major players in the cycles of main nutrients like nitrogen (a major compound for proteins), phosphorous (an element essential for energy transfer) and carbon (the fundamental element of life on Earth). Most species with unicellular endosymbiotic cyanobacteria (*Synechococcusspongiarium* complex) show a red, brown, or purple external coloration in life. Examples in this checklist include *Neofibularianolitangere*, *Aplysina*spp., *Verongula*spp., *Ircinia*spp., *Geodia*spp., *Erylus*spp., *Neopetrosia*spp., *Petrosia*spp., *Xestospongia*spp., etc. Sponges play a well-known role in reef accretion by gluing the reef framework (Wulff and Buss 1979) or by generating a structurally complex hard substrate, and in bioerosion, coral skeletons, and other calcium carbonate substrates (by species of *Cliona* and *Siphonodictyon*). Few studies have evaluated the dimension, diversity, or dynamics of these sponge roles at mesophotic depths. Therefore, this is an open and exciting horizon to explore, discover, and quantify through the diverse and extensive sponge community in the northwestern Gulf of Mexico and elsewhere.

## Supplementary Material

XML Treatment for Agelas cf. citrina

XML Treatment for Agelas clathrodes

XML Treatment for Agelas dilatata

XML Treatment for Ptilocaulis cf. walpersii

XML Treatment for Myrmekioderma gyroderma

XML Treatment for Ectyoplasia ferox

XML Treatment for Didiscus oxeatus

XML Treatment for Higginsia coralloides

XML Treatment for Biemna cribaria

XML Treatment for Neofibularia nolitangere

XML Treatment for Acanthella cubensis

XML Treatment for Acanthella cf. mastophora

XML Treatment for Auletta tuberosa

XML Treatment for Auletta syncinularia

XML Treatment for Auletta

XML Treatment for Scopalina ruetzleri

XML Treatment for Placospherastra antillensis

XML Treatment for Haliclona

XML Treatment for Callyspongia (Cladochalina) cf.armigera

XML Treatment for Neopetrosia proxima

XML Treatment for Petrosia

XML Treatment for Petrosia weinbergi

XML Treatment for Xestospongia muta

XML Treatment for Xestospongia

XML Treatment for Niphates erecta

XML Treatment for Siphonodictyon

XML Treatment for Siphonodictyon brevitubulatum

XML Treatment for Batzella rubra

XML Treatment for Batzella cf. rubra

XML Treatment for Clathria

XML Treatment for Halichondria

XML Treatment for Topsentia bahamensis

XML Treatment for Rhizaxinella clava

XML Treatment for Corallistes typus

XML Treatment for Neophrissospongia cf. nolitangere

XML Treatment for Stellettinopsis cf. megastylifera

XML Treatment for Erylus alleni

XML Treatment for Erylus goffrilleri

XML Treatment for Erylus trisphaerus

XML Treatment for Geodia cf. curacaoensis

XML Treatment for Discodermia

XML Treatment for Yucatania sphaeroidocladus

XML Treatment for Cinachyrella

XML Treatment for Chondrosia collectrix

XML Treatment for Aiolochroia crassa

XML Treatment for Aplysina

XML Treatment for Aplysina cf. archeri

XML Treatment for Verongula rigida

XML Treatment for Verongula reiswigi

XML Treatment for Ianthellide

XML Treatment for Aplysilla aff. sulfurea

XML Treatment for Dysidea

XML Treatment for Pleraplysilla

XML Treatment for Pleraplysilla

XML Treatment for Ircinia campana

XML Treatment for Ircinia cf. campana

XML Treatment for Ircinia strobilina

XML Treatment for Ircinia

XML Treatment for Ircinia

XML Treatment for Smenospongia cf. echina

XML Treatment for Plakortis cf. zyggompha

XML Treatment for Dactylocalyx pumiceus

XML Treatment for Iphiteon panicea

## Supplementary materials

Supplementary material 1 Table of localities for mesophotic sponge species in the North Western Gulf of Mexico Data type occurrences, geographic coordinates, habitat type, and localities Explanation note The table summarizes distribution data for sponge species collected and observed at 17 localities at the Flower Garden Banks National Marine Sanctuary Region. https://binary.pensoft.net/file/848740This dataset is made available under the Open Database License (http://opendatacommons.org/licenses/odbl/1.0/). The Open Database License (ODbL) is a license agreement intended to allow users to freely share, modify, and use this Dataset while maintaining this same freedom for others, provided that the original source and author(s) are credited.Maria Cristina Díaz, Marissa Nuttall, Shirley A. Pomponi, Klaus Rützler, Sarah Klontz, Christi Adams, Emma L. Hickerson, G.P. Schmahl
